# Oxygen Variability in the Offshore Northern Benguela Upwelling System From Glider Data

**DOI:** 10.1029/2022JC019063

**Published:** 2022-11-05

**Authors:** Elisa Lovecchio, Stephanie Henson, Filipa Carvalho, Nathan Briggs

**Affiliations:** ^1^ National Oceanography Centre European Way Southampton UK

**Keywords:** oxygen, hypoxia, mesoscale, upwelling, Benguela, eddy

## Abstract

Despite their role in modulating the marine ecosystem, variability and drivers of low‐oxygen events in the offshore northern Benguela Upwelling System (BenUS) have been rarely investigated due to the events' episodicity which is difficult to resolve using shipboard measurements. We address this issue using 4 months of high‐resolution glider data collected between February and June 2018, 100 km offshore at 18°S. We find that oxygen (O_2_) concentrations in the offshore northern Benguela are determined by the subsurface alternation of low‐oxygen Angola‐derived water and oxygenated water from the south at 100–500 m depth. We observe intermittent hypoxia (O_2_ < 60 μmol kg^−1^) which occurs on average for ∼30% of the 4 months deployment and is driven by the time‐varying subsurface pulses of Angola‐derived tropical water. Hypoxic events are rather persistent at depths of 300–450 m, while they are more sporadic and have weekly duration at shallower depths (100–300 m). We find extreme values of hypoxia, with O_2_ minima of 16 μmol kg^−1^, associated with an anticyclonic eddy spinning from the undercurrent flowing on the BenUS shelf and showing no surface signature. Fine‐scale patchiness and water mass mixing are associated with cross‐frontal stirring by a large anticyclone recirculating tropical water into the northern BenUS. The dominance of physical drivers and their high variability on short time scales reveal a dynamic coupling between Angola and Benguela, calling for long‐term and high‐resolution measurements and studies focusing on future changes of both tropical O_2_ minima and lateral fluxes in this region.

## Key Points

- Alternating subsurface physical fluxes of Angola‐ and Benguela‐derived waters drive mesopelagic oxygen (O_2_) variability in the offshore Benguela
- Hypoxic events (O_2_ < 60 μmol) are intermittent and are associated with weekly pulses of tropical water in the upper mesopelagic
- Mesoscale eddies further modulate the O_2_ field and are associated with severe hypoxia, fine‐scale O_2_ patchiness and water mass mixing

## 1 Introduction

Ocean oxygen (O_2_) concentrations influence the distribution and functioning of marine life and are determined by a combination of physical and biological mechanisms (Fennel & Testa, 2019; Garçon et al., 2019; Pitcher et al., 2021). The near‐surface ocean is generally well oxygenated: there, O_2_ levels are mostly regulated by a combination of temperature‐dependent and fast air‐sea exchange fluxes balancing O_2_ super‐ or undersaturation and biological activity, especially primary production in the euphotic layer, which adds O_2_ via photosynthesis (Sarmiento & Gruber, 2006). Below the euphotic layer, advection and mixing (both horizontal and vertical) and biological activity in the form of remineralization and respiration dominate the O_2_ budget. While respiration always acts as a sink for O_2_, contributing to the formation of deep O_2_‐minima, physical fluxes can either increase or decrease O_2_ concentrations depending on their source region (Wyrtki, 1962). Ocean ventilation, that is, the deep injection of well‐oxygenated surface waters via mixing or advection, acts to replenish the deep O_2_ budget. Lateral fluxes, upwelling and small scale variability connected to meso‐ and submesoscale eddies and fronts further modulate the O_2_ field by influencing a variety of biological processes, which in turn modify O_2_ concentrations (Brandt et al., 2015; Frenger et al., 2018; Löscher et al., 2016).

Variability in ocean O_2_ concentrations has been extensively studied in the past decades, especially in relation to the expansion of the oxygen minimum zones (OMZs) driven by anthropogenic forcing (Ito et al., 2017; Pitcher et al., 2021; Schmidtko et al., 2017). Spatio‐temporal changes in O_2_ concentration can constitute significant stressors for marine ecosystems and can have potentially lethal effects on important fisheries (Grantham et al., 2004; Ohde & Dadou, 2018). Most marine heterotrophs, including higher animals such as fish, die when exposed to O_2_ concentrations below a certain critical threshold, which varies significantly among species (Keeling et al., 2010; Vaquer‐Sunyer & Duarte, 2008). Even intermittent occurrences of such lethal O_2_ levels can result in behavioral changes due to the need to frequently escape such conditions, and can result in declining growth rates in juvenile fish (Eby et al., 2005). The median lethal time in low‐oxygen conditions also varies greatly from species to species, and ranges from several months to as little as half an hour (Vaquer‐Sunyer & Duarte, 2008). As a consequence, low O_2_ levels effectively constitute a biogeochemical boundary for most marine species and can strongly affect the ecosystem composition and predator/prey interactions (Gibson & Atkinson, 2003).

Among the regions most affected by deoxygenation, eastern boundary upwelling systems (EBUS) are extremely significant (Garçon et al., 2019; Karstensen et al., 2008; Löscher et al., 2016), as they bound some of the ocean's major OMZs while also hosting high levels of coastal productivity that supports some of the most important fisheries for human sustainment (Carr & Kearns, 2003; Kainge et al., 2020; Mackas et al., 2006). The Benguela Upwelling System (BenUS), one of four major EBUS, is located along the south‐western African coast, at the eastern edge of the South Atlantic gyre, between roughly 18°S and 34°S (Chavez & Messié, 2009; Hutchings et al., 2009; Sowman & Cardoso, 2010). As with all EBUS, the BenUS is characterized by high levels of mesoscale and submesoscale activity in the form of surface and subsurface intensified eddies, filaments and fronts, which have an important role in modulating marine O_2_ levels via a variety of biophysical interactions (Frenger et al., 2018; McGillicuddy, 2016; Mohrholz et al., 2014; Thomsen, Kanzow, Colas, et al., 2016).

**Figure 1:**
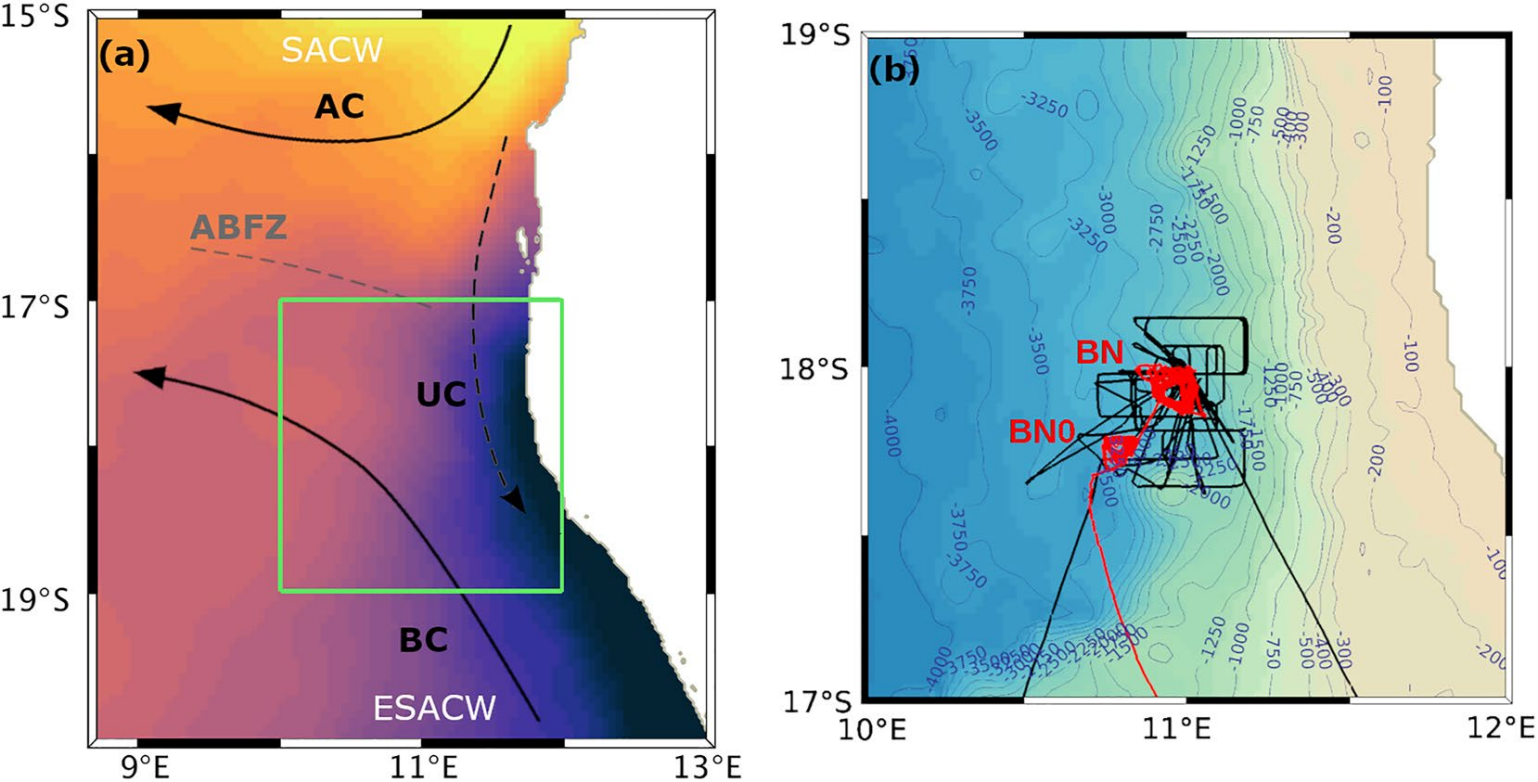
(a) Schematic representation of the regional system of currents and of the water masses carried by them, as described by the literature presented in the Introduction: Angola current, Benguela current, Angola‐Benguela frontal zone, cross‐front undercurrent (UC), South Atlantic Central Water, Eastern South Atlantic Central Water. This scheme is a simplification of the regional current schemes by Rouault et al. (2007), Hardman‐Mountford et al. (2003) and Siegfried et al. (2019). This is only a conceptual scheme which does not intend to be an exact representation of the system of currents nor of the location of their maxima. The green square indicates the perimeter of subplot (b). Color shading: sea surface temperature from L4 Advanced Very‐High‐Resolution Radiometer reprocessed Ostia (Good et al., 2020) averaged across the glider measurement period. (b) Glider (red) and ship (black) tracks on the regional GEBCO (2019) topography [m]. Labels indicate the spots Benguela North 0 and Benguela North in which the glider collected data.

At its northern edge, the BenUS is bounded by the Angola‐Benguela frontal zone (ABFZ), which separates the system from the equatorial region of Angola and is located roughly between 15° and 18°S (Veitch et al., 2006). The ABFZ is characterized by significant meridional gradients of physical and biogeochemical tracers, including temperature, salinity and O_2_, which reflect the different properties of the water masses located at its northern and southern edges (Bartholomae & van der Plas, 2007; Kostianoy & Lutjeharms, 1999; Veitch et al., 2006). North of the ABFZ, the equatorial cyclonic circulation of Angola is dominated by the warm, saline and O_2_‐depleted South Atlantic central water (SACW) and hosts the most intense OMZ of the Atlantic ocean (Bartholomae & van der Plas, 2007; Karstensen et al., 2008; Stramma et al., 2008). South of the ABFZ, the BenUS is crossed by the along‐shore northward‐flowing Benguela current (BC), which carries relatively colder, fresher and oxygenated eastern South Atlantic central water (ESACW), a blend of equatorial SACW and Indian central water transported into the South Atlantic by the Agulhas Current (Mohrholz et al., 2008; Siegfried et al., 2019). At the ABFZ, the northward‐flowing BC and the southward‐flowing Angola Current (AC) converge along the coast and are deflected westward toward the open ocean, with a slight northward tilt (Figure 1b). Variability in the temperature and salinity of the region surrounding the ABFZ and in the latitudinal location of the front can be determined by interannual fluctuations known as Benguela Niños/Niñas as well as seasonal and intraseasonal shifts in the atmospheric wind pattern (Diakhaté et al., 2016; Kostianoy & Lutjeharms, 1999; Veitch et al., 2006).

Along‐shore fluxes recurrently cross the ABFZ (Boyer et al., 2000; John et al., 2000). At the surface, the AC can intrude into the northern BenUS along the shelf in the shape of a tongue of surface warm water, especially in December–March (Bartholomae & van der Plas, 2007; Muller et al., 2014; Shannon et al., 1987); at depth, a subsurface flow of Angola water extends far into the Benguela region with maximum intensity in January–February (Mohrholz et al., 2008; Rouault, 2012; Stramma & England, 1999). Such cross‐frontal fluxes happen as a result of both wave propagation and local wind forcing (Tchipalanga et al., 2018). Equatorially forced coastal trapped waves that flow along the continental shelf transporting Angola water southward constitute a mode of coupling between the equatorial region and the BenUS and can transfer variability ranging from the sub‐seasonal to interannual scales (Bachèlery et al., 2020; Illig & Bachèlery, 2019; Kopte et al., 2017). Coastal‐trapped waves forced by local winds can further modulate the shelf flow on the faster scales of a few days to weeks, either enhancing or stopping the propagation of the poleward shelf current (Junker et al., 2019). Further connection between the tropical circulation and northern Benguela is provided by the south‐eastward flow of the equatorial currents (Siegfried et al., 2019). The along‐shore advection of low‐oxygen anomalies from the north has been shown to be the primary driver of O_2_ variability on the shelf of the northern BenUS (Monteiro et al., 2006). Local remineralization can further exacerbate these conditions (Ohde & Dadou, 2018).

So far, most studies have focused on the O_2_ variability on the BenUS shelf, with only a limited analysis of the impact on the adjacent open waters off the northern Benguela shelf. Further, observations focusing on the short‐term fluctuations in the O_2_ concentration and the impact of mesoscale variability in such a dynamic region are still lacking. Here we use 4 months of high‐resolution glider data collected 100 km from the BenUS coast to investigate the temporal variability of O_2_ concentrations in the open waters off the shelf of the northern BenUS, focusing on significant fluctuations observed in the upper mesopelagic (100–500 m) and their relation to the dominance of SACW and ESACW. Further, we discuss the duration, intensity and depth distribution of the hypoxic events, and present two distinct examples of how meso‐ and submesoscale activity shapes O_2_ concentrations in our data. Last, we put our results in the context of previous research in order to identify the physical drivers of the observed variability and present our conclusions.

## 2 Methods

### 2.1 Glider Data

One Slocum glider was deployed on 14 February 2018 at 11.2°E, 19.3°S in the region of the northern BenUS (Figure 1a) by the European Research Council project Gauging ocean Organic Carbon fluxes using Autonomous Robotic Technologies, in conjunction with the Controls over Ocean Mesopelagic Interior Carbon Storage (COMICS) program (Sanders et al., 2016). The glider was deployed 3 months ahead of the COMICS cruise (which occurred in May–June 2018) in order to characterize the water column dynamics and temporal variability in this region. The glider transited north for 3 days from its deployment location to the sampling site Benguela North 0 (BN0) (10.80°E, 18.25°S), where it profiled to 1,000 m depth from 17 February 2018 until 26 March 2018. On 27 March 2018, the glider moved slightly north‐east to measurement point Benguela North (BN), centered around (10.95°E, 18.05°S), where it sampled until 19 June 2018, when it was recovered. The two measurement spots are located at about 120 km (BN0) and 95 km (BN) from the south‐western African coast and are separated from each other by roughly 27 km. Both locations are off the continental shelf, north of the Walvis Ridge, in a region with maximum bathymetry of about 3,000 m.

At each sampling location, the glider followed a triangular path of ∼12 km per side which took between 2 days in February‐May to 5 days in June to complete. The glider completed profiles to 1,000 m depth, sampling with a vertical resolution of ∼20 cm, emerging 5 to 6 times per day and sampling primarily on the upward dive. A few sets of consecutive downcast‐upcast were collected throughout the deployment to allow the determination of O_2_ sensor time response. The glider was fitted with a standard Slocum Glider Payload conductivity, temperature, and depth (CTD) (pumped) from Seabird (SN 9109), measuring Temperature, Conductivity and Depth, with an Aanderaa optode, model 4831 (SN286), measuring dissolved O_2_, and a Seabird WetLABS Environmental Characterization Optics (ECO) triplet sensor, measuring optical backscattering at 700 nm. Depth‐averaged currents (DAC) were estimated for each full 1,000 m downward and upward dive from the difference between the glider's observed surfacing location and that predicted by the glider's onboard dead reckoning or flight model (Merckelbach et al., 2010; SLOCUM manual, 2017). For each glider surfacing, glider surface currents were also estimated via linear regression of global positioning system (GPS) location with respect to time. Median timescale of surface current estimates, defined as the interval between first and last GPS fix was 21 min with 95% between 10 and 48 min. It is worth noticing that glider estimates of surface currents may be subject to a wind influence as the glider tail may acts temporarily as a sail in strong wind conditions.

In the present analysis, conservative temperature (T_*c*_, from here on: temperature), absolute salinity (S_*a*_, from here on: salinity), potential density (*σ*_*θ*_) and dissolved O_2_ glider data are used in the form of binned medians, with each bin representing an interval of 6 hr in time (usually 1–2 profiles) and 2 m in depth. Glider surface and DAC velocities were averaged daily. Backscatter data (b_bp_700) are averaged on coarse bins spanning 1 day in time and 10 m in depth. Please, note that in this manuscript backscatter data is used only qualitatively to strengthen some of our hypothesis and is only presented in the Supplement. As the glider measurements were collected during 2018, we will implicitly refer to this year throughout the rest of the manuscript.

Glider temperature and salinity values were initially calculated using factory calibrations. Next, lag‐corrected O_2_ data were calculated by applying the following steps: (a) calculate sensor time response, determined by shifting the reported O_2_ in time and find the time lag that corresponds to the minimum total discrepancy between consecutive profiles (up‐down and down‐up) of O_2_ (50 s for this glider); (b) apply time lag correction to raw sensor output “calphase”; (c) calculate O_2_ concentration using the coefficients from factory multi‐point calibration; (d) apply temperature, salinity and pressure correction following manufacturer recommendations. Finally, shipboard CTD profiles collected during COMICS cruise DY090 were used to validate glider temperature and salinity measurements and adjust glider O_2_ measurements, in a two‐step process. First, salinity and dissolved O_2_ sensors mounted on the ship's CTD package were calibrated using linear regressions against 113 bottle salinity measurements (using an Autosal 8400B) and 261 bottle O_2_ measurements (using the Winkler titration method). Second, all shipboard and glider sensor profiles were binned at 5 m depth intervals and “ship‐glider matchups” were selected for glider calibration, defined as binned profiles within 5 km and 12 hr of each other. These matchups were strongly correlated (linear regression *r* ^2^ ≥ 0.95). Paired binned data from all matchups (*n* = 18 for temperature, *n* = 17 for salinity, and *n* = 16 for O_2_, where *n* is the number of CTD casts) were combined to calculate a single linear regression of ship data versus glider data. Glider temperature and salinity data matched ship data within uncertainty of the regressions and were therefore not corrected. Glider O_2_ data were corrected using the regression slope (1.03) and offset (+1.72 μmol kg^−1^) found in the calibration exercise (see Figure S14 in Supporting Information S1). Gliders measured optical backscattering at 700 nm using Seabird ECO triplet sensors. Factory calibrations were applied to raw data to yield estimates of the total volume scattering function *β*(700 nm, 124°). The volume scattering function of seawater was estimated following Hu et al. (2019) and subtracted to yield the particulate volume scattering function *β*_p_(700 nm, 124°) and then converted to the particulate backscattering coefficient b_bp_(700 nm) via b_bp_(700 nm) = *β*_p_(700 nm, 124°) 2*πχ*, where *χ* = 1.077 (Sullivan et al., 2013).

### 2.2 Water Mass Identification

The glider measurements were collected at the northern boundary of the BenUS (Figure 1a), in the proximity of the ABFZ. We identify the two central water masses in the glider data by converting the SACW and ESACW water mass boundary values of Mohrholz et al. (2008); Poole and Tomczak (1999); Flohr et al. (2014) for in‐situ temperature T_*i*_ and practical salinity S_*p*_ into units of absolute salinity (S_*a*_) and conservative temperature (T_*c*_) using the TEOS‐10 Gibbs Seawater Oceanographic Toolbox (McDougall & Barker, 2011). We acknowledge that the conversion from T_*i*_ and S_*p*_ to T_*c*_ and S_*a*_ is non‐linear. However, we used these points to qualitatively assess the presence of the two water masses in the glider data by plotting the line that connects the extreme values of T_*c*_ and S_*a*_ for each water mass, which is sufficient for our purposes. Please refer to Table S1 in Supporting Information S1 for the details of the conversion and the adopted values of T_*c*_ and S_*a*_.

We also used World Ocean Atlas 2018 (WOA18) data (NOAA data set, 2018; Garcia et al., 2019) to compare the glider absolute salinity and O_2_ data to the long‐term mean regional profiles. Salinity from WOA18 was transformed into units of absolute salinity (S_*a*_) using the tools described in the previous paragraph. Mean profiles were calculated using the 1° gridded product for the offshore Angola waters north of the glider in the range (9°E, 11°E) × (15°S, 17°S) and for the offshore Benguela waters south of the glider in the range (10°E, 12°E) × (21°S, 23°S). Mean WOA18 profiles were calculated from climatological (1955–2017) monthly means from February to June. Given the strong variability and sharp lateral gradients that characterize the measurement region, this analysis provides more information than comparing the glider data with sporadic measurements or coarse resolution products around the glider location.

### 2.3 Glider Data Analysis: Tracer Anomalies and Stratification

In order to highlight the time variability in the tracer concentrations in the glider data, we calculated tracer anomalies from their mean vertical profiles over the entire period of sampling. Although this does not remove the seasonal trends in the glider data, we found this choice to be more appropriate than the use of a running mean, due to the limited temporal extent of the measurements and sharp horizontal and vertical gradients found by the glider. Further, this choice allows us to explore both the short scale and the lower frequency variability in the tracers. We therefore take into account both the role of small scale variability and the role of seasonality in the discussion of the tracer anomalies.

The mixed layer depth (MLD) was calculated according to a temperature criterion, as the deepest depth at which *T* = *T*_10*m*_ ± 0.8°C (Kara et al., 2003). As a measure of water column stability, we calculated the Brunt‐Väisälä frequency, defined as:

(1) $$N^{2}=-\frac{g}{\rho_{0}}\cdot \frac{\partial \sigma_{\theta }}{\partial z}$$

where $\frac{\partial \sigma_{\theta }}{\partial z}$ is the derivative of potential density with respect to depth, g is gravitational acceleration and *ρ*_0_ = 1,026 kg m^−3^ is the mean reference density of seawater, corresponding to our average density in the most dynamic first 400 m depth.

### 2.4 Regional Setting During the Measurement Campaign

We used several data products to identify the large‐scale physical and biogeochemical context in which the glider measurements were taken. The evolution of currents, sea surface temperature (SST) and chlorophyll (CHL) concentration were evaluated from daily mean satellite data. We ran the algorithm by Faghmous et al. (2015) on each time step of the Archiving, Validation and Interpretation of Satellite Oceanographic (AVISO) data sea surface height (SSH) field (CMEMS, 2022) to identify the eddies, each one defined as the area within the outermost closed SSH contour containing a single SSH minimum (cyclonic eddy) or maximum (anticyclonic eddy). SST was taken from the L4 Advanced Very‐High‐Resolution Radiometer (AVHRR) reprocessed Ostia data set (Good et al., 2020), consisting of gap‐free maps combining high‐resolution data in cloud free conditions (1/20° resolution) and lower resolution images (1/4° resolution). Daily mean surface CHL was taken from the L3 GlobColour data set (CMEMS, 2020) at a spatial resolution of 4 km.

We used the in‐situ glider estimates of near surface currents and DAC, as well as ship, satellite and reanalysis products to evaluate the near‐surface flow at the glider position. Ship measurements of near‐surface velocities (30 m depth) were collected using a shipboard acoustic doppler current profiler (Teledyne RD Instruments Acoustic Doppler Current Profiling (ADCP) Ocean Surveyor, 75 KHz) at measurement point BN from 02 June 2018 until the end of the measurement campaign on 19 June 2018 (Figure 1a), and they were averaged daily for the present analysis. Additional gridded products were co‐located with the glider using a nearest‐neighbor routine that calculates a weighted average of the velocities at the closest four grid points from the glider on each day. We used daily mean AVISO and GlobCurrent satellite data at 1/4° resolution (CMEMS, 2022; Rio et al., 2014). Both data sets are based on the same SSH‐derived geostrophic currents, however GlobCurrent uses additional modeled Ekman transport to better capture the nearshore wind‐driven dynamics. Note that the AVISO product often underestimates the westward wind‐driven currents, resulting at times in excessive eastward flow, for example, in early April when the AVISO surface flow results exceeds all the other products; GlobCurrent, however, at times seems to overestimate the westward (off‐shore) transport in disagreement with the observed SST and CHL fields, especially in late April. A comparison of the two fields is provided in the Supplement (Figure S3 in Supporting Information S1). On cloud‐free days, surface currents at 30 km resolution were estimated by spatially correlating consecutive images of L2 surface CHL data from Moderate Resolution Imaging Spectroradiometer (MODIS) Aqua, MODIS Terra, and Visible Infrared Imaging Radiometer Suite (VIISRS) at 1 km resolution (NASA‐OBPG, 2018a; NASA‐OBPG, 2018b, 2018c) via the maximum cross correlation method (Crocker et al., 2007; Liu et al., 2017); daily fields were calculated as the median of the available surface velocity data for each day. We also used daily mean surface velocities from the GLORYS12v1 reanalysis product (Drévillon et al., 2018; Fernandez & Lellouche, 2018) based on the Operational Mercator global ocean analysis and forecast system which uses the physical model Nucleus for European Modelling of the Ocean (NEMO) forced with ERA5 reanalysis data (Hersbach et al., 2020; Madec & Team, 2022) on a 8 km‐resolution grid. A list of the employed data sets is provided in Table S2 in Supporting Information S1.

### 2.5 Upwelling Indexes

We calculated the wind‐driven upwelling components using daily mean wind stress from ERA5 reanalysis (Hersbach et al., 2020) at 1/4° resolution. To calculate the coastal upwelling index (*U*_*i*_), we rotated the wind stress (*τ*) components in the alongshore (AS) direction, averaged it in the first 100 km from the coast, and then used:

(2) $$U_{i}=\frac{\tau_{AS}}{f\rho }$$

where *f* is the latitude‐dependent Coriolis parameter and *ρ* is the density of seawater (same as Subsection 2.3). *U*_*i*_ was averaged in the latitude range (17°S, 19°S), in the proximity of the glider. To calculate Ekman pumping, we calculated the curl of the wind stress and derived the Ekman pumping velocity *w_EKP_*:

(3) $$w_{\mathrm{EKP}}=\frac{\nabla \times \vec{\tau }}{f\rho }$$

from which we obtained the pumping velocity at the glider position *w_gl_* by selecting the grid point at (11°E, 18°S). To obtain the nearshore Ekman pumping index *W*_*i*_ we calculated the volumetric transport via an area integral of *w_EKP_* in first 100 km from the coast in the range (17°S, 19°S) and divided this by the coastline length in the same range. This way both upwelling indexes are in units of volumetric water transport per meter of coastline (m^2^ s^−1^). Following Messié et al. (2009), to calculate W_*i*_ we set negative values *w_EKP_* to zero before integration, to reflect the asymmetry in the transport of nutrients.

### 2.6 Low Oxygen Thresholds

Throughout the manuscript, we study low O_2_ events during which concentrations fall below a set of three thresholds: O_2_ < 120 μmol kg^−1^; O_2_ < 60 μmol kg^−1^, here defined as hypoxia; and finally O_2_ < 30 μmol kg^−1^, here defined as severe hypoxia. These thresholds are intended to highlight a set of critical O_2_ conditions for different marine organisms. Our loosest threshold of 120 μmol kg^−1^ is used to represent the mean sub‐lethal O_2_ threshold for fish (triggering behavioral and metabolic changes) as well as the lethal threshold for sensitive crustacean and fish larvae (Ekau et al., 2010; Vaquer‐Sunyer & Duarte, 2008). Our hypoxic threshold (60 μmol kg^−1^) closely represents the mean lethal O_2_ concentration calculated across a wide range of marine species, and is commonly adopted for studies of the impact of low O_2_ events on fisheries (Gilly et al., 2013; Vaquer‐Sunyer & Duarte, 2008; Zhang et al., 2010). Severe hypoxia (O_2_ < 30 μmol kg^−1^) indicates critical conditions for some low O_2_‐resistant organisms such as gelatinous plankton, squids and copepods (Ekau et al., 2010). When evaluating the depth range affected by low O_2_ anomalies for each threshold, we exclude regions in which the anomalies are observed for less than 1% of the deployment period.

## 3 Results

### 3.1 Regional Setting

**Figure 2:**
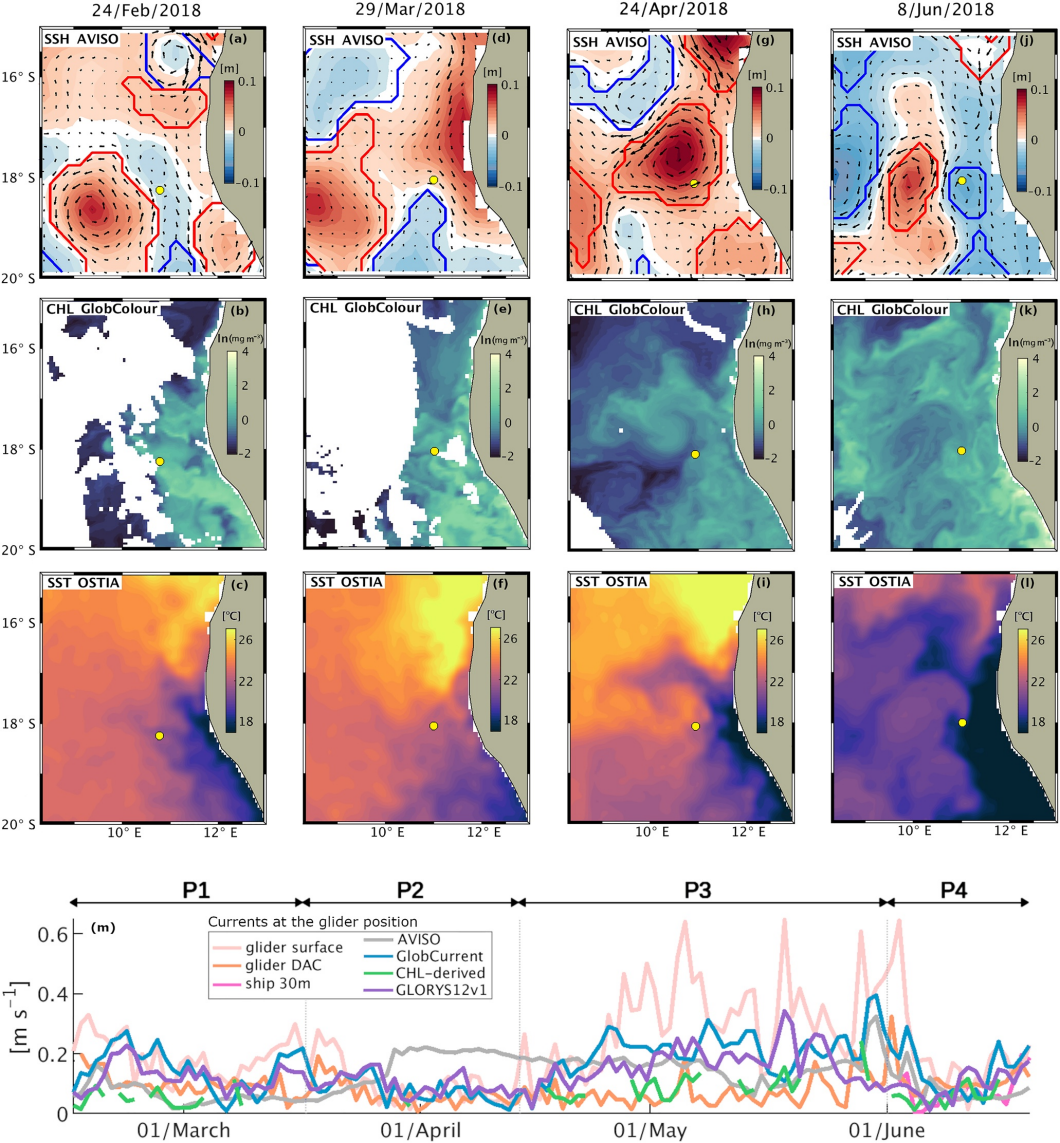
Regional setting from satellite data on 24/February (a–c), 29/March (d–f), 24/April (g–i), 08/June (j–l). First row: sea surface height (SSH) [m] with geostrophic velocities from Archiving, Validation and Interpretation of Satellite Oceanographic data (AVISO). Second row: surface chlorophyll (CHL) [ln(mg m^−3^)] concentration from GlobColour. Third row: sea surface temperature (°C) from L4 Advanced Very‐High‐Resolution Radiometer ‐Ostia. Line contours on SSH indicate the identified cyclonic (blue) and anticyclonic (red) eddies. White areas in CHL images indicate missing data due to cloud cover. The mean daily glider position is indicated by a yellow dot. (m) Horizontal velocities (absolute) at the glider position from satellite data (AVISO, GlobCurrent), satellite CHL derived data (CHL‐derived), model reanalysis data (GLORYS12v1), glider data both at the surface (glider surface) and depth‐averaged (glider DAC), and ship data for velocities at 30 m depth. The duration of phases P1 to P4 is indicated by the horizontal arrows above subplot (m), each one also corresponding to the satellite images in the column above it.

The glider data were collected during February–June 2018, that is, during the period spanning from the low upwelling season to the early phase of the winter upwelling season of the northern Benguela (Hutchings et al., 2009). SST anomalies for 2018 indicate neutral Benguela Niño conditions (Imbol Koungue et al., 2021). Satellite images and horizontal velocity products (Figure 2) show a succession of very different conditions in the region surrounding the glider. We identify four periods (P1–P4), each one characterized by a specific type of dynamics. Phase 1 (P1: 14 February–16 March) is characterized by moderate currents at the glider position, by a cold water signature along the BenUS coast and by a complex regional pattern of high‐CHL filaments; the glider is located north‐east of a large and persistent anticyclone and surface satellite images suggest that the glider is hit from south‐east by cold and high CHL coastal water. Phase 2 (P2: 17 March–13 April) is characterized by minimum surface flow at the glider position and by a tropical warm water intrusion along the Benguela shelf visible as a narrow low‐CHL and warm SST anomaly reaching down to 19°S, which temporarily reverses the cross‐shore SST gradient. Phase 3 (P3: 14 April–31 May) is characterized by the formation (late April), intensification (early May) and drifting/dissipation (late May) of a large frontal anticyclone at the ABFZ (see Figures S1, S2 in Supporting Information S1). During this period horizontal currents at the glider position intensify, peaking in early May when the glider is located at the outer edge of the anticyclone in a region characterized by sharp gradients of CHL, while coastal upwelling pulses and large fluctuations in the zonal and meridional currents indicate strong winds and turbulence. Phase 4 (P4: 01 June–19 June) is characterized by blooming conditions and a cooling of the SST likely due to a combination of pulses of upwelling in early June and the transition toward Austral winter. The glider is embedded in the cold‐water front originating at the Benguela shelf and including a weak cyclonic eddy.

### 3.2 Water Column Properties From the 2D Glider Transects

**Figure 3:**
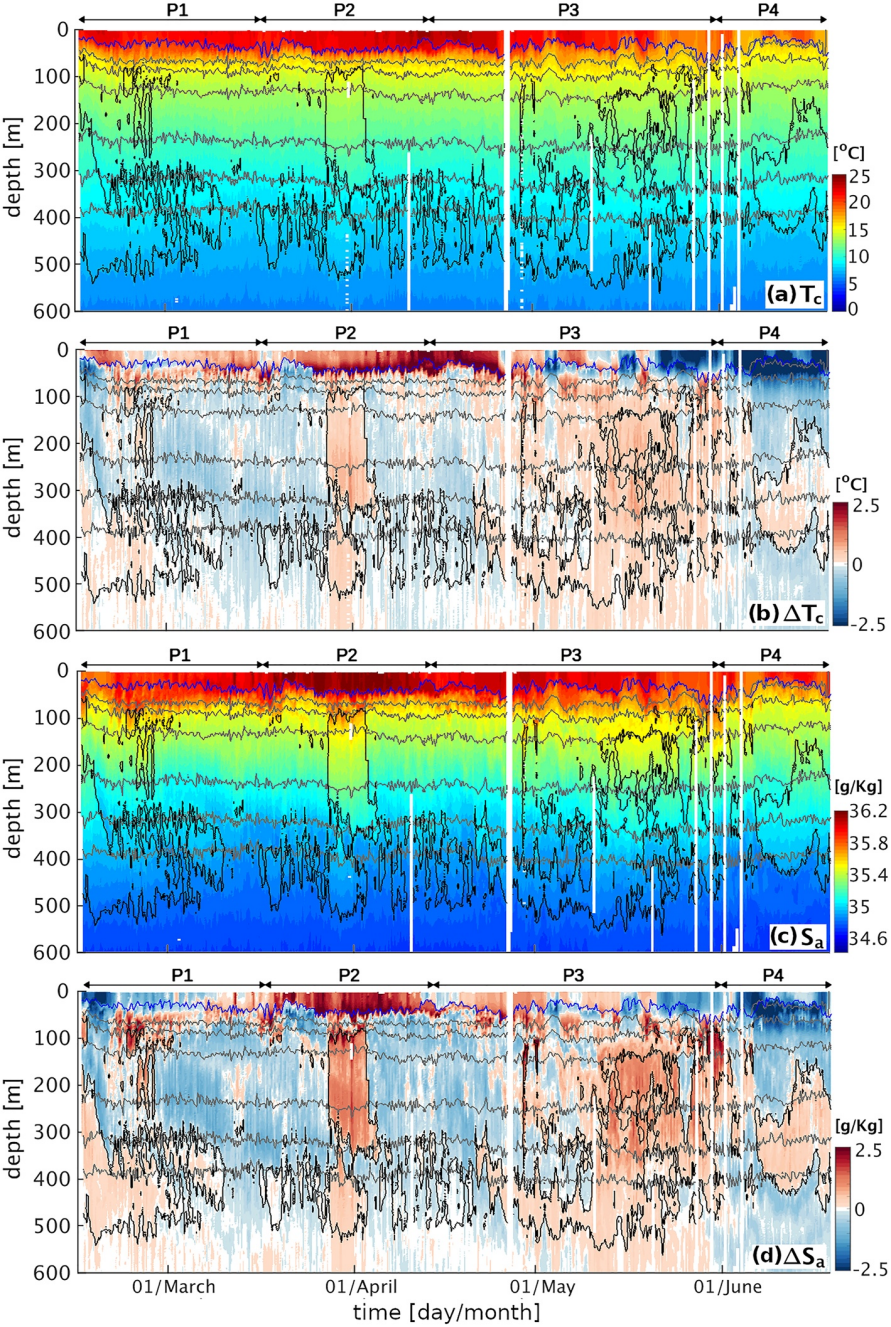
Glider transects up to 600 m depth: (a) conservative temperature, (b) conservative temperature anomaly, (c) absolute salinity, (d) absolute salinity anomaly. Gray lines: isopycnals for 26, 26.25, 26.5, 26.75, 26.9, 27 (kg m^−3^). Black lines: outer boundary of hypoxic regions (O_2_ = 60 μmol kg^−1^). Blue line: MLD [m]. Full profiles to 1,000 m depth are available in the Supplement (Figures S5, S6 in Supporting Information S1).

The glider data show clear fluctuations in the tracer distributions at all depths to at least 500 m depth (Figure 3). The water column was rather stratified, with a mean MLD of 35 m, rarely exceeding 50 m. The distinct timescales of variability of near‐surface and deeper water properties suggests some degree of decoupling between these layers and a shift in the dominant drivers of tracer fluctuations, with an abundance of deep water T_*c*_ and S_*a*_ anomalies that are not reflected by the surface fields.

At depths of 50–500 m, water properties are very dynamic, characterized by alternating positive and negative anomalies in temperature and salinity. In this range of depths, in fact, temperature and salinity are characterized by a qualitatively similar pattern of positive and negative anomalies that alternate each other, lasting for periods that range between a few days and a few weeks. Some of the short timescale variability in the transects, persisting throughout the entire data series and generating sub‐weekly oscillations, is likely associated with the lateral movement of the glider around the sampling triangle, and is therefore a signature of spatial, not temporal, gradients. Below 500 m depth, tracer oscillations are more moderate and persist for longer periods than in the upper layers, suggesting a further shift of the variability drivers from the more dynamic upper mesopelagic region (see Figures S5, S6 in Supporting Information S1 for the full depth transects).

**Figure 4:**
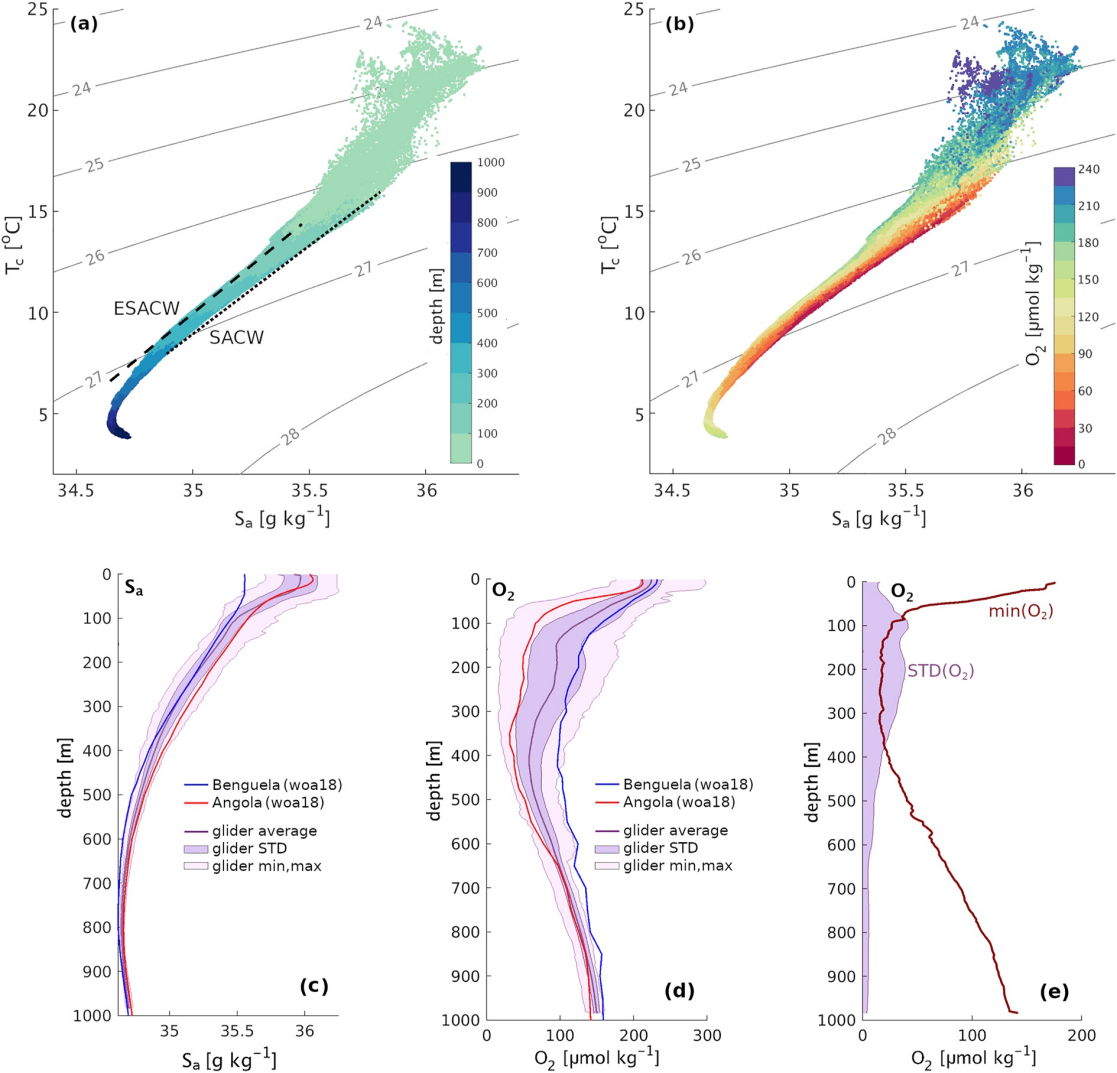
(a, b) Conservative temperature (T_*c*_) and absolute salinity (S_*a*_) diagram of the binned glider data, with density isolines in gray (kg m^−3^) and dots colored according to: (a) depth, (b) oxygen (O_2_) concentration. (a) The characteristic T_*c*_‐S_*a*_ trends of South Atlantic central water and eastern South Atlantic central water are highlighted respectively by a dotted and a dashed black line. (c), (d) Mean profile, standard deviation range and minimum‐maximum range for (c) absolute salinity and (d) O_2_ from the glider data (purple line and shadings) compared to climatological mean profiles across the period February–June for the offshore Angola (red lines) and Benguela (blue lines) from World Ocean Atlas 2018 (see Methods). (e) Depth profile of the standard deviation and minimum of O_2_ from glider data.

The temperature and salinity diagram of the sampled water masses shows that the known T_*c*_‐S_*a*_ curves of SACW and ESACW constitute the extreme boundaries of the central water masses measured by the glider (Figure 4a). As expected given the known characteristics of the two water masses, points located on the SACW line are characterized by very low O_2_ concentrations, often falling below 60 μmol kg^−1^, while those located in correspondence with ESACW are characterized by O_2_ concentrations of at least 100 μmol kg^−1^ (Figure 4b). Such T_*c*_‐S_*a*_ diagrams and the observed fluctuations in physical properties point toward a co‐presence of water from the northern Angola and from the southern latitudes of the BenUS at the glider position, with the two water masses alternating each other between 100 and 500 m depth. This result is further emphasized by a comparison of the vertical S_*a*_ and O_2_ profiles at the glider position with the climatological mean vertical profiles of the offshore Angola and Benguela waters (Figures 4c and 4d), showing a remarkable correspondence between the climatological profiles and the range of values measured by the glider. Interestingly, a large number of points fall between the SACW and ESACW lines in both our T_*c*_‐S_*a*_ diagrams and in the vertical profile plots, therefore suggesting that part of the two water masses mix with each other in the region of sampling.

### 3.3 Oxygen Variability and Its Relation to Water Properties

**Figure 5:**
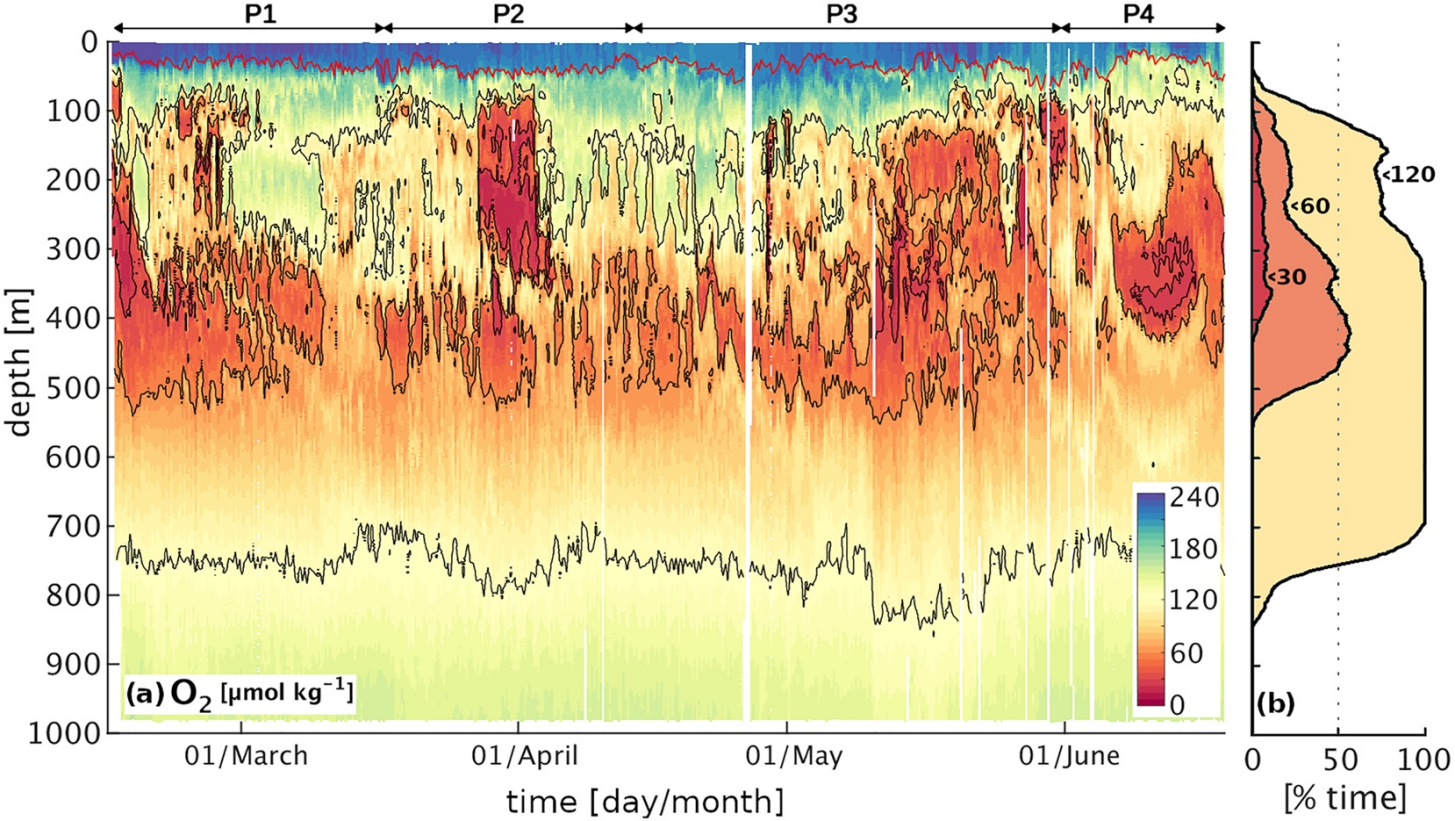
(a) Oxygen (O_2_) transect from glider data with black isolines of concentration at 30, 60, 120 μmol kg^−1^ and mixed layer depth in red; (b) percent of total time in which O_2_ is below 30 μmol kg^−1^ (red), 60 μmol kg^−1^ (orange) and 120 μmol kg^−1^ (yellow) as a function of depth.

Glider data show that O_2_ concentrations drop significantly across the first 100 m depth (Figure 5). The MLD is characterized by values above 200 μmol kg^−1^ and constitutes the only near‐surface range where O_2_ consistently exceeds the sublethal threshold of 120 μmol kg^−1^. The mean O_2_ concentration drops below this threshold at 110 m depth and O_2_ concentrations exceeding sublethal levels are observed sporadically at larger depths. At around 100 m depth, minimum O_2_ values also drop quickly to just 25 μmol kg^−1^ (Figure 4e).

**Figure 6:**
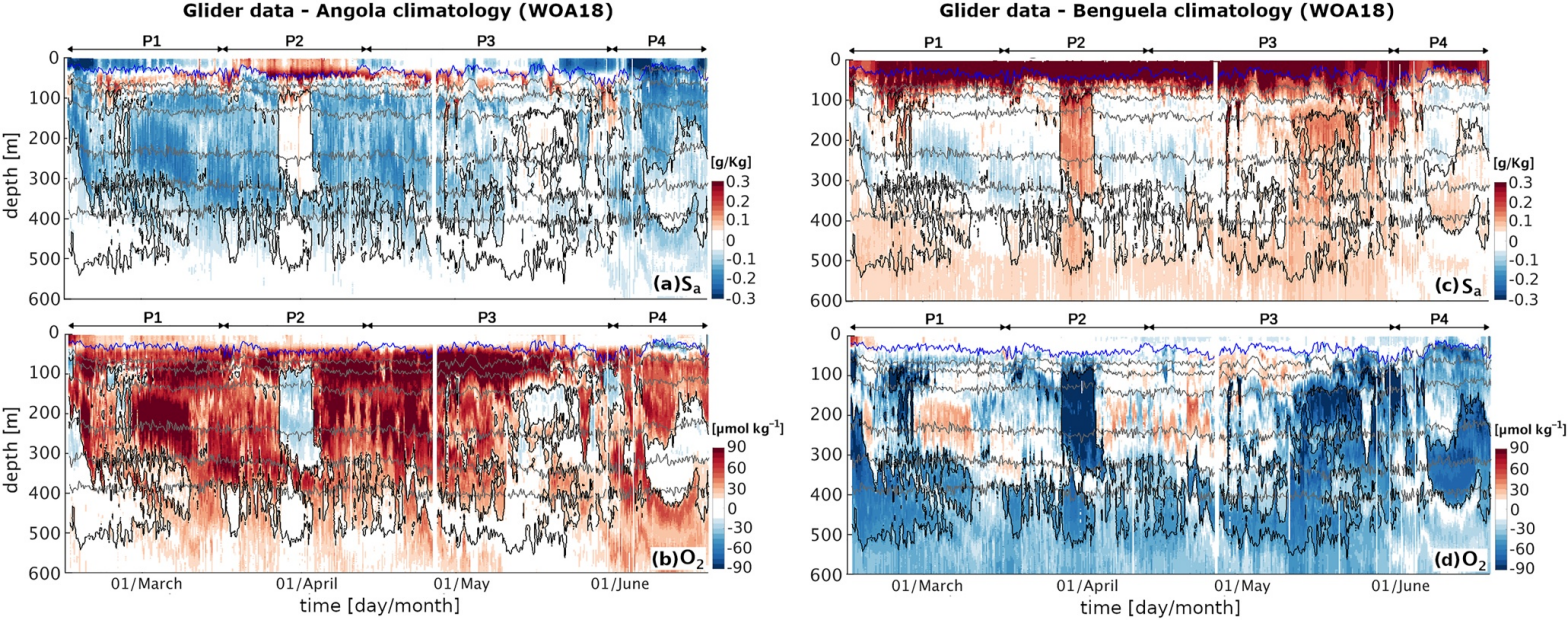
Difference between the glider data transects and the climatological mean profiles across the period February‐June for the offshore Angola (a), (b) and offshore Benguela (c), (d) water properties (see Methods). (a), (c) Absolute salinity S_*a*_ difference and (b), (d) oxygen O_2_ concentration difference. White shading covers the range (−0.05, 0.05) kg m^−3^ for S_*a*_, and (−15, 15) μmol O_2_m^−3^ for O_2_. Gray lines: isopycnals for 26, 26.25, 26.5, 26.75, 26.9, 27 (kg m^−3^). Black lines: outer boundary of hypoxic regions (O_2_ = 60 μmol kg^−1^). Blue line: MLD [m]. Full profiles up to 1,000 m depth are available in the Supplement (Figures S8, S9 in Supporting Information S1).

The temporal variability in the O_2_ concentration increases significantly below the MLD (Figure 5a), where it closely follows the pattern of ΔT_*c*_ and ΔS_*a*_. This shift is due, on the one hand, to the lack of air‐sea exchange fluxes and a limited impact of biological production below the near‐surface layer, and, on the other hand, to the significant role of the deep lateral influx of northern warm/saline/O_2_‐depleted SACW and southern cold/fresh/oxygenated ESACW, as highlighted by the association of positive temperature and salinity anomalies with negative O_2_ anomalies. This interpretation is further supported by a comparison in time between the sampled salinity and O_2_ profiles and the climatological mean salinity and O_2_ properties of the waters found north of the glider (offshore Angola) and south of the glider (offshore Benguela), showing a remarkable match between salinity and O_2_ properties with the two water masses (Figure 6). As a result, hypoxic events in the upper mesopelagic at the glider position can be attributed to the dominance of saline and de‐oxygenated water from the northern latitudes, while higher O_2_ levels are carried into the region by fresher central waters from southern Benguela latitudes.

Although local remineralization likely further modulates the O_2_ profile at the glider position, our analysis highlights that the dominant drivers of the sub‐monthly O_2_ variability within 100–500 m depth are physical, resulting in weekly to bi‐weekly hypoxic events associated to shifts in temperature and salinity. Importantly, this high variability implies that sporadic observations are unlikely to capture the average O_2_ profile in this region, likely sampling temporary fluctuations around the mean. Further, a decoupling between the observed variability of the near‐surface and of the deeper water properties suggests that the signature of these alternating fluxes of water masses may not always be detectable from the surface. Indeed, evidence of a southward transport in the satellite data emerges clearly only on the shelf during P2 and at the glider position in relation to the anticyclonic recirculation of tropical water across the front during P3 (Figure 2).

### 3.4 Oxygen Variability: Intermittency of Hypoxia

**Figure 7:**
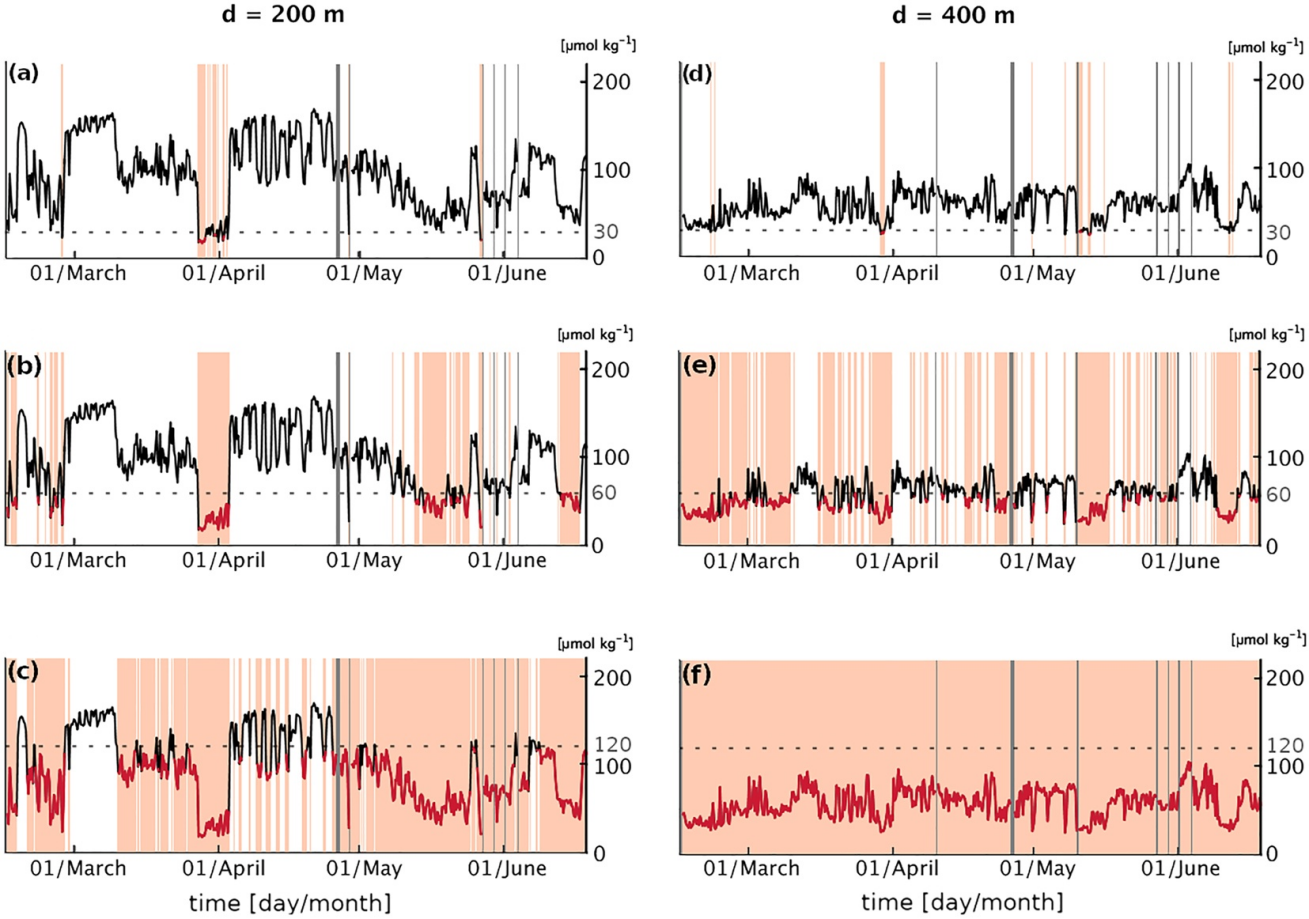
Time variability and duration of the low oxygen (O_2_) events at two different depths of reference, one per column for the three O_2_ thresholds evaluated: (a–c) 200 m, ∼22% probability of occurrence of hypoxia; (d–f) 400 m, ∼54% probability of occurrence of hypoxia. Orange shading highlights when O_2_ falls below a certain threshold, indicated by the horizontal dashed gray line: (a), (d) 30 μmol kg^−1^; (b), (e) 60 μmol kg^−1^; (c), (f) 120 μmol kg^−1^. Gray shading indicates missing data.

As a consequence of the alternating dominance of SACW and ESACW, both hypoxia and severe hypoxia observed in the upper mesopelagic are intermittent throughout the entire measurement period (Figure 7). Hypoxic events (O_2_ < 60 μmol kg^−1^) are confined between ∼70 m and ∼550 m depth, where they occur for an average of ∼30% of the deployment, increasing to ∼50% within the sub‐range of 300 and 450 m depth (Figure 5b). Severe hypoxic events, in contrast, are shallower and more confined vertically, being observed only between ∼110 m and ∼420 m and occurring on average for ∼6% of the total measurement time. Severe hypoxic events ranged in timescale from <1 day to ∼1 week, with the largest single event occurring in the beginning of April (P2), when severe hypoxia was observed just below 100 m depth (Figure 5). More sporadic severe hypoxia is also detected at deeper depths by the glider during the early (P1) and late measurement period (P3–P4), the latter following some bursts of coastal upwelling (see Figure S2 in Supporting Information S1).

In order to correctly interpret the results we must keep in mind the spatio‐temporal nature of the observed fluctuations. As the glider was repeatedly visiting the same locations while moving along a triangular path of 12 km of side, the observed variability is due to a combination of temporal changes in the dominant water mass and spatial gradients, the latter generating oscillations with a period of 2 days during February‐May and 5 days in June, when the glider slowed down. Sub‐weekly fluctuations in O_2_ concentration and salinity (Figure 5) therefore indicate that the glider was moving across sharp gradients of O_2_, highlighting spatial patchiness in O_2_ and sharp gradients at the region of sampling. Weekly and persistent fluctuations, on the other hand, are associated with larger scale shifts in the pattern of water masses at the glider position and therefore on significant changes in the pattern of currents intersecting the region of sampling.

Our data shows that, in the depth range where hypoxia is most persistent (300–450 m depth) the glider‐derived mean O_2_ and salinity profiles are closer to the climatological profiles of the Angola latitudes (Figures 4c and 4d), suggesting that the presence of subsurface SACW is more persistent at these depths. On the contrary, in the shallower depth range between 100 and 300 m, where we can identify weekly and bi‐weekly pulses of hypoxia and intense salinity anomalies, the glider O_2_ and salinity profiles show a large standard deviation and fall just between the Angola and Benguela profiles (Figure 4), indicating a higher degree of temporal variability in the influence of SACW and ESACW. Interestingly, a few of the low O_2_ anomalies, such as the early April severe hypoxic event, show de‐oxygenation below the mean levels observed in the Angola waters located north of the glider (Figure 6), despite maintaining a match in salinity with these waters, possibly indicating a different history of biogeochemical transformations of the associated water.

### 3.5 Oxygen Variability: Mesoscale Drivers

We focus here on P2 and P3, illustrating two distinct mesoscale processes captured by the glider data and affecting the O_2_ distribution in the offshore northern BenUS. These also correspond to some of the shallowest and most intense hypoxic events observed during the glider campaign.

#### 3.5.1 Phase 2: Severe Hypoxia Associated With the Undercurrent Intensification and the Likely Spinning of a Subsurface Eddy

**Figure 8:**
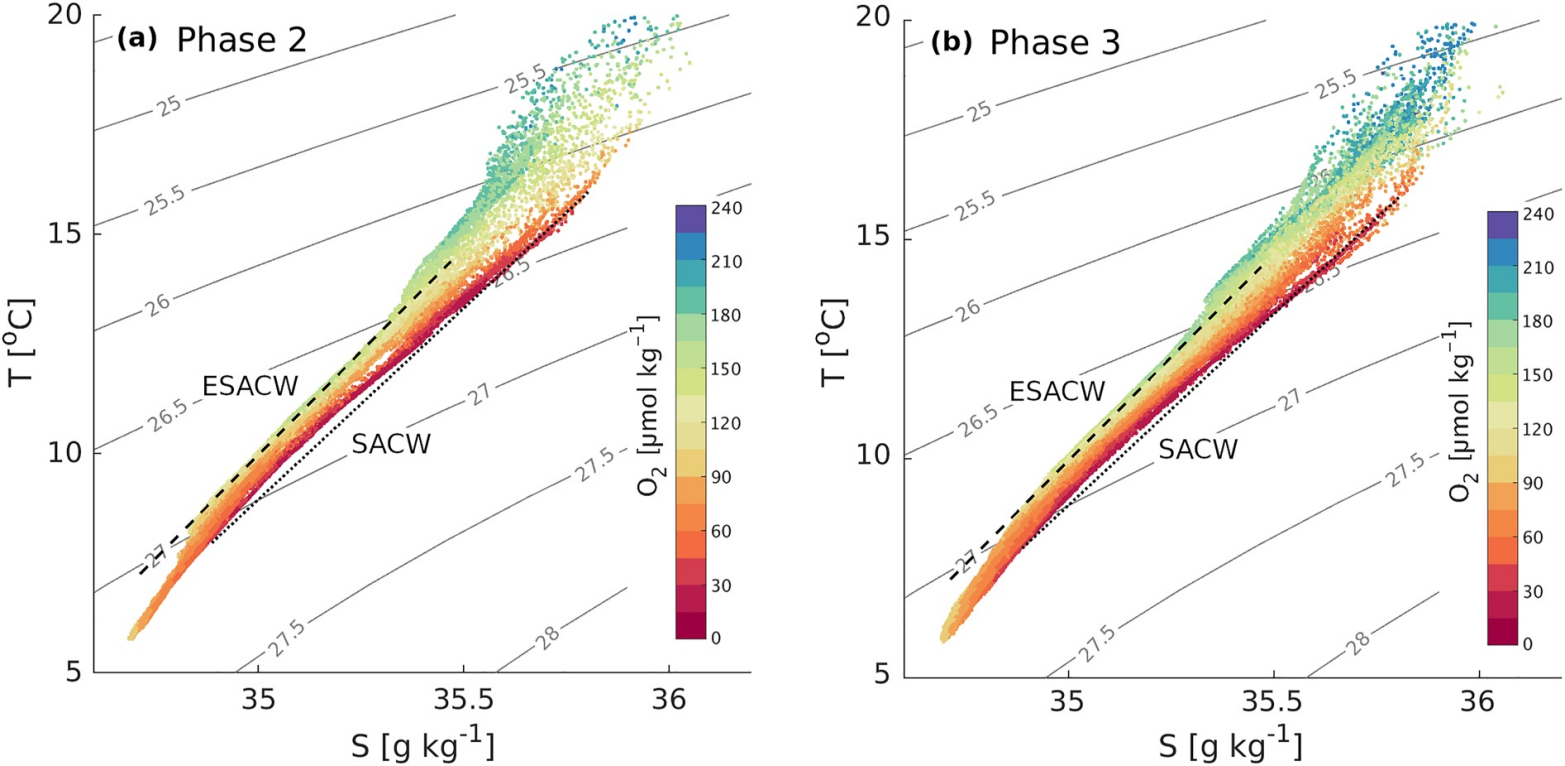
Conservative temperature (T_*c*_) and absolute salinity (S_*a*_) diagram of the binned glider data restricted to the range of 50–600 m depth during (a) phase 2 (17 March–13 April), and (b) phase 3 (16 April–31 May). Dots are colored according to oxygen concentration, density isolines are plotted in gray (kg m^−3^). The characteristic T_*c*_‐S_*a*_ trends of South Atlantic central water and eastern South Atlantic central water are highlighted respectively by a dotted and a dashed black line. Full‐depth diagrams for the 4 phases can be found in Figure S12 in Supporting Information S1.

P2 (17 March–13 April) is characterized by visible surface poleward flow along the BenUS shelf and by minimum lateral flow at the glider position (Figures 2d–2f, 2m). Despite the relatively calm surface water conditions, a sharp and persistent hypoxic event spans the depth range between 75 and 300 m for about 1 week between 27 March (when the glider reaches measurement spot BN) and 03 April, hosting significant domains of severe hypoxia (Figure 5, P2). In addition to concurrent positive temperature and salinity anomalies, the hypoxic event is associated with a deep bulging of the isopycnals (Figure 3, P2), and a local minimum in the Brunt‐Väisälä frequency (N^2^), especially between 200 and 300 m depth (see Figures S7c, S10d in Supporting Information S1). This set of properties is characteristic of subsurface anticyclonic eddies, previously observed in upwelling regions and often shed by the shelf undercurrents carrying low O_2_ waters (Frenger et al., 2018; Molemaker et al., 2015). As satellite SST for P2 indicates the presence of an active poleward transport of warm and potentially deoxygenated tropical water along the BenUS shelf (Figure 2f), this hypothesis is especially likely. We must note that the N^2^ minimum is not as clear as expected for a sub‐surface eddy structure, and it shows only a limited signature of a sharp peripheral maximum that would indicate the lateral isolation of the eddy core. This potentially indicates that the detected structure may still be unstable or in formation (see further discussion in Subsection 4.2). The T*c*–S*a* diagram for P2, however, shows a clear separation between hypoxic and oxygenated waters (Figure 8a), indicating a sharp transition between SACW and ESACW and therefore weak mixing between water masses, which is not observed in the other phases (Figure 8b, see also Figure S12 in Supporting Information S1).

We therefore infer that the detected structure is most likely a subsurface anticyclone spinning from the undercurrent. This structure combines extreme hypoxic conditions with a lack of a surface signature, which makes it impossible to detect from satellite data. Further, the limited lateral mixing between the water within and outside of the structure, as indicated by the separation of water masses in the T_*c*_‐S_*a*_ diagram for P2, results in a sharp but transitory pattern of hypoxia and severe hypoxia. Minimum O_2_ concentrations in this structure recurrently fall below 20 μmol kg^−1^ and reach as low as 16 μmol kg^−1^. Such O_2_ concentrations are lower than the climatological mean observed for the water mass located north of the glider (Figure 6, P2); this anomaly also corresponds to a dip in particle concentrations (see Figure S7a in Supporting Information S1, P2). Both signatures suggest that, compared to the surrounding waters, this water was subject to higher levels of remineralization, possibly occurring on the shelf. The largest spatial gradients at the edge of the anomaly were found between 200 and 400 m depth, with O_2_ concentrations varying by between 30 and 40 μmol kg^−1^ km^−1^. Given that the glider was sampling along a triangle of 12 km per side, the diameter of the structure must have been larger than about 16 km in order to be sampled continuously for a week's time—however, we are unable to estimate the full size of the structure and therefore to evaluate whether it was in the meso‐ or submesoscale range.

#### 3.5.2 Phase 3: Stirring at the Rim of a Large Frontal Anticyclone

During P3 (16 April–31 May), the glider is located in a region of intense lateral surface flow, that is, at the rim of a large anticyclone that stirs northern offshore Angola water and southern Benguela water across the ABFZ (Figures 2g–2i, 2m; Figure S1 in Supporting Information S1). Satellite images show that from late April to mid‐May (eddy formation and intensification phases) the glider crosses fronts between northern warm&low‐CHL water and cold&high‐CHL water from the nearshore Benguela. This emerges as strong variations in physical properties both within the mixed layer and at depth (Figure 3, P3), and in O_2_ concentrations at depths of 100–500 m (Figure 5, P3). Oxygen within the mixed layer, instead, is dominated by fast air‐sea exchange fluxes. A fine pattern of concurrent fluctuations in T_*c*_, S_*a*_ and O_2_ persists across May, especially at depths of 100–500 m, indicating that the glider is crossing very sharp gradients between water masses. During this period, spatial gradients in the O_2_ concentrations exceed 50 μmol kg^−1^ km^−1^. These small scale patterns and the lack of separation between water masses shown by the T_*c*_‐S_*a*_ diagram for P3 (Figure 8b) suggests that eddy stirring is enhancing mixing between water masses. This is also suggested by the difference plots between S_*a*_ and O_2_ and the water mass properties north and south of the glider, which also show intermediate salinity and O_2_ concentrations in early May (Figure 6, P3).

In the second half of May, the eddy detaches from the coast to move offshore (see Figure S1 in Supporting Information S1). Glider data show a progressive switch from a prevalence of ESACW to SACW, and fronts start to be associated with sporadic severe hypoxia at depths of 250–450 m, a fine pattern that is not as shallow nor as persistent as the one observed during P2. Mid to late May also sees an intensification of coastal upwelling with intense bursts in late May–early June (see Figure S2a in Supporting Information S1), corresponding to O_2_ concentrations falling below the climatological mean of the northern water mass at depths of 100–200 m (Figure 6, P3–P4). The fine‐scale pattern observed in late May is likely to result from a combination of anticyclonic stirring, upwelling intensification bringing low‐oxygen shelf water toward the glider position and a reactivation of the AS poleward transport of SACW subsequent to the offshore drift of the eddy.

## 4 Discussion

### 4.1 Physics Driving Oxygen Variability in the Offshore Northern Benguela

The results of our manuscript show that, at the latitudes of the measurements, physical processes in the form of alternating lateral fluxes of SACW and ESACW are the dominant drivers of sub‐monthly fluctuations in the O_2_ availability between 100 and 500 m depth in the offshore northern Benguela. Hypoxia at the glider location can't be explained only by a combination of sluggish ventilation and local biological uptake of organic material. These observations extend to offshore waters the findings of previous studies highlighting that physical processes, in the form of lateral fluxes of hypoxic SACW waters, are the primary driver of hypoxia and the main trigger of the onset of anoxia (near‐zero O_2_ concentrations) on the shelf of the BenUS (Monteiro et al., 2006, 2008). Although anoxia is not observed in our data set, both hypoxia and severe hypoxia emerge from the lateral influx of SACW, as suggested by the analysis of the T_*c*_‐S_*a*_ signatures and a comparison with the climatological offshore Angola and Benguela O_2_ and salinity profiles. More oxygenated conditions, on the other hand, are associated to an inflow of intermediate waters with T_*c*_‐S_*a*_ signature characteristic of the southern‐derived ESACW. The observed magnitude and temporal scale of variability in O_2_ also suggests that data sets aiming to resolve monthly means and long‐term O_2_ trends in the offshore northern Benguela should account for such sub‐monthly fluctuations and make sure to adopt a statistically significant set of measurements in order not to incur in biases. Continuous and long‐term observations are of great value to account for the observed variability and to correctly resolve the monthly mean O_2_ profile in the open waters of the northern BenUS.

The signatures of SACW and ESACW at 100–350 m depth alternate with each other on the timescale of one to 2 weeks at the glider position, while SACW seems more persistent at deeper depths. Only some of these anomalies can be associated with mesoscale features. Modeling studies by Muller et al. (2014); Schmidt and Eggert (2016) show that, in austral summer, the shelf undercurrent at 18°S deepens to about 200–400 m depth in the offshore region of the system. Most of the observed fluctuations may therefore be associated with short‐lived pulses of the shelf undercurrent reaching the offshore region of the northern Benguela and carrying water from the north, which may determine an intensification and/or wider depth range of the poleward transport. Variability in temperature, salinity and O2 on the scale of a few days to 2 weeks was also observed by Monteiro et al. (2006) on the BenUS shelf at 23°S. The O2 variability on the shelf and in the subsurface off‐shore northern BenUS region may therefore be modulated on similar temporal scales. Studies showed that coastal trapped waves generated by wind stress forcing within the BenUS oscillate with a period of a few days to weeks (Junker et al., 2019), and may therefore contribute to the variability of the transport of SACW along the shelf at the latitude of the glider.

### 4.2 Mesoscale Drivers

On top of the large‐scale circulation, mesoscale activity impacts O_2_ concentration at the glider position. We identify two mesoscale processes that affect the O_2_ variability at the time of sampling: lateral advection of severely hypoxic water most likely by a subsurface anticyclone during an intensification of the undercurrent (P2), and cross‐frontal stirring by a large anticyclone (P3). While the structure associated with P3 is identifiable from surface satellite data, this is not the case for P2. Since glider measurements at BN were collected at roughly 95 km from the BenUS coast, a subsurface eddy spinning from the shelf undercurrent is likely to be young or in formation at such a limited offshore distance (Thomsen, Kanzow, Krahmann, et al., 2016) and possibly still unstable, which could explain the weak N^2^ signature. This is also suggested by observing the lateral extension of multiple forming anticyclonic loops in model reanalysis data during the undercurrent intensification of P2 (see GLORYS12v1 reanalysis data in Figure S4 in Supporting Information S1). It is also unlikely that the glider sampled continuously at the very center of the eddy core, resulting in a higher degree of noise in N^2^. A detailed analysis of the glider position for each profile indicates that the glider may have entered and exited the structure from the same southern side, and moved along the triangular sampling path within its perimeter for the duration of the anomaly. Alternative hypotheses, such as the anomaly being associated with poleward laminar transport by the undercurrent flowing off the shelf rather than an eddy spinning off the shelf portion of the current, would not be able to explain the shape of the isopycnals nor the especially low O_2_ and particle concentrations of P2 compared to the other observed hypoxic events (Figure 6b and Figure S7a in Supporting Information S1). Anticyclonic eddies shed by subsurface currents, in fact, are known to trap and advect extremely low O_2_ shelf water (Frenger et al., 2018), while high remineralization rates in well‐isolated subsurface eddy cores can further exacerbate low O_2_ conditions (Karstensen et al., 2015, 2017). The anomaly being associated to a precursor of an actual eddy, such as instabilities in the flow of the subsurface current (Molemaker et al., 2015), is also unlikely due to the anomalies suggesting some degree of coherence and isolation in the structure. Small scale laminar transport from the coast, for example, upwelling filament transport, cannot explain the sluggish flow observed during the anomaly in velocity products (Figure 2m) nor the deep signature of the structure, as filaments are typically confined to the upper 100 m (Cravo et al., 2010; Thomsen, Kanzow, Colas, et al., 2016).

Surface and subsurface eddies are an intrinsic mode of flow in upwelling systems (Chaigneau et al., 2009; Frenger et al., 2018). The mesopelagic O_2_ field in the sampled region is therefore recurrently impacted by their associated O_2_ signature, fine scale patchiness and water mass mixing. Subsurface eddies in the BenUS were previously detected in Argo temperature and salinity profiles (McCoy et al., 2020), however, to our knowledge, hypoxia within a subsurface eddy shedding from the BenUS undercurrent has not previously been observed in situ. There is an abundance of literature studying the physical properties and biogeochemistry of such eddies in other upwelling systems based on in‐situ and model data (e.g., Chaigneau et al., 2011; Schütte, Karstensen, et al., 2016; Fiedler et al., 2016; Karstensen et al., 2017; Frenger et al., 2018; Auger et al., 2021) which have found that low‐oxygen anomalies trapped and further intensified within subsurface eddies generated along the shelf are transported toward the middle of the gyre, where they shape the large‐scale O_2_ gradient of mid‐latitudes. In the tropical North Atlantic, a satellite‐based study estimated these structures constituted 9% of the eddy population (Schütte, Brandt, & Karstensen, 2016). However, additional preliminary work based on models suggests a remarkably higher ratio of subsurface eddies globally (40% of all the eddies), with most (75%) showing no surface signature (Schütte et al., 2021, talk), therefore suggesting an even more significant impact on biogeochemical fields.

Large frontal eddies such as the one observed in P3 are also known to be a recurrent feature of the ABFZ (Boyer et al., 2000). The fine‐scale T_*c*_, S_*a*_ and O_2_ patterns (Figures 3 and 5) and the intermediate salinity and O_2_ properties (Figures 6 and 8) observed in mid May indicate that such cross‐frontal anticyclones are an effective means through which SACW and ESACW mix at the ABFZ and that, as a result, such eddies smooth the cross‐frontal tracer gradients. From the perspective of the Angola region, this lateral stirring is likely to ventilate the South Atlantic OMZ by laterally advecting and stirring higher O_2_ concentrations into the northern latitudes, similar to what is observed for example, in the North Atlantic (Kolodziejczyk et al., 2018).

### 4.3 Seasonality and Future Trends

Seasonality is expected to play a role in our results. Glider measurements were collected between February and June, that is, during the low upwelling season characterized by minimum O_2_ levels on the BenUS shelf and frequent intrusions of tropical water (Boyer et al., 2000). With the onset of the austral winter‐spring upwelling season, more oxygenated and nutrient‐rich waters are brought onto the shelf (Mohrholz et al., 2008; Monteiro et al., 2008), possibly resulting in less frequent, weaker and/or more confined hypoxic events at the measurement site. Although our glider measurements do not fully capture seasonal variability, the data show changes in all tracers approaching June, that is, toward the beginning of winter (Figures 3 and 5). With the onset of upwelling, during late P3 and P4 (see Figure S2a in Supporting Information S1), glider data show that low O_2_ anomalies are confined within a narrower range of depths, between 200 and 400 m. During the upwelling season, the offshore BenUS may therefore be characterized by even sharper O_2_ gradients between different depths. Phases characterized by active upwelling (late P3–P4 and, to a lesser extent, P1) also present some severe hypoxic concentrations, although the signals are not as persistent as during P2. In the case of shallow and short‐lived severe hypoxic events such as the one emerging at the very end of P3, this may be possibly linked to some low‐oxygen shelf water being advected offshore toward the glider. Longer lasting severe hypoxic events observed during the upwelling intensification, such as the one observed during June (P4) between 300 and 400 m, are deeper than the one detected during P2 and therefore fall close to the climatological O_2_ profile of the northern SACW (Figure 6b). Longer data series and model studies are needed in order to better decouple the role of the southward and offshore transport of low O_2_ water during periods of intensified upwelling. Longer term data are also needed to collect enough statistics of these O_2_ fluctuations and understand how seasonal changes in currents and biological activity affect their duration and depth distribution.

This study further highlights the strong lateral coupling between the biogeochemistry of Angola and Benguela, which extends beyond the shelf region into the open waters. The recent and future expansion of the ocean's OMZs, including the Angola OMZ, constitutes a significant risk for ecosystems and fisheries in upwelling regions (Bograd et al., 2023; Bopp et al., 2013; Keeling et al., 2010). As O_2_ levels fall, hypoxic events will become more probable, shrinking the habitable range for many marine species (Breitburg et al., 2018; Gilly et al., 2013). Observations suggest that in the equatorial Atlantic O_2_ concentrations have largely declined in the last decades, falling by about −5 μmol kg^−1^ at a depth of 300 m over the last 50 years (Schmidtko et al., 2017). Lower O_2_ levels in the Angola region are likely to impact the offshore northern BenUS, with pulses of undercurrent likely transporting increasingly deoxygenated shelf waters and frontal eddies stirring more severe low O_2_ anomalies into more southerly latitudes, and possibly also across a wider range of depths. Furthermore, our results highlight that the impact of large scale changes in O_2_ concentrations on the offshore northern Benguela will depend also on the future evolution of the physical fluxes coupling the this region with the southern Benguela latitudes and with the Angola. Eddy‐resolving coupled physical‐biological model simulations are needed to better understand these trends and their potential impact on the local ecosystem.

### 4.4 Implications for Marine Organisms

Our results provide information on the extension of the habitable depth range for different marine species in the offshore northern BenUS and can provide useful data to better understand their metabolic and behavioral adaptation to the local O_2_ variability and the spatial patchiness and temporal intermittency of hypoxia. Hypoxic events (O_2_ < 60 μmol kg^−1^) during our deployment period were confined between 68 and 546 m depth, with a probability of occurrence exceeding 10% at 100 m depth, that is, just beneath the most productive layer. These events, although intermittent in time, constitute significant stressors for marine organisms and can strongly modulate the ecosystem behavior (Eby et al., 2005; Gilly et al., 2013; Keppel et al., 2016; Vaquer‐Sunyer & Duarte, 2008). Spatial and temporal patchiness in oxygenation can create niches for hypoxia‐resistant organisms as well as force sensitive species to migrate, thus modulating the coupling between different trophic levels, and potentially forcing prey closer to their predators or predators away from their prey (Bell & Eggleston, 2005; Breitburg, 2002). Since severe hypoxia is confined to a depth range narrower than that of hypoxia, organisms that can tolerate very low O_2_ levels, such as copepods, gelatinous plankton and squids, have the potential to occupy a larger habitable zone not only in the near surface but also at depth (Ekau et al., 2010; Gilly et al., 2013). Sub‐lethal O_2_ levels (O_2_ < 120 μmol kg^−1^), on the other hand, become increasingly more probable with depth up to ∼700 m depth. This implies that organisms that are extremely sensitive to low O_2_, such as crustaceans and fish larvae, are likely confined within the mixed layer, while many fish species sensitive to sub‐lethal O_2_ thresholds in this range may alter their metabolism in order to inhabit a wider range of depths (Vaquer‐Sunyer & Duarte, 2008).

## 5 Conclusions and Outlook

In the present study we discussed the temporal variability of O_2_ concentrations in the offshore northern BenUS using high‐resolution glider data collected between February and June 2018. Our results show that weekly alternating fluxes of tropical SACW and southern ESACW are the primary driver of the significant O_2_ fluctuations in the upper mesopelagic. Hypoxic events are connected to the presence of tropical water and are intermittent in time, rarely exceeding 1 week's duration in the range of 100–300 m depth, revealing significant variability in the pattern of subsurface fluxes at the ABFZ. Further, we demonstrated the key role of the mesoscale subsurface transport from the shelf and mesoscale lateral stirring across the ABFZ in driving severe hypoxia, subsurface O_2_ patchiness and water mass mixing at the Angola‐Benguela front.

Our results underline the importance of continuous and high‐resolution observations in the offshore region of the northern Benguela, in order to better characterize the observed O_2_ variability and correctly resolve its mean. Longer time series of in‐situ data and modeling studies are needed to clarify how frequency, duration, intensity and spatial patterns of both hypoxic events and the driving SACW/ESACW subsurface fluxes vary with seasons and on interannual time scales. The role of local biology in further modulating the detected low O_2_ anomalies should also be clarified, including addressing the consequences for particle concentrations and export during hypoxic events. The effect of intermittent hypoxia on organisms and ecosystem structure of upwelling regions also needs further investigation (Eby et al., 2005; Keppel et al., 2016), especially with regards to diel vertical migrators, which might be particularly impacted by the observed subsurface advection of hypoxic water. Further, modeling studies can also help to understand how hypoxia variability and prevalence in the northern BenUS will evolve in the context of future changes as a result of the region's lateral coupling with both the South Atlantic OMZ and the southern Benguela (Breitburg et al., 2018; Gilly et al., 2013).

## Conflict of Interest

The authors declare no conflicts of interest relevant to this study.

## Supporting information

Supporting Information S1

## Data Availability

The binned glider data used for this study can be downloaded at: https://doi.pangaea.de/10.1594/PANGAEA.938221 Other publicly available datasets such as satellite data and model reanalysis data used for this study have been listed in detail in Table S2 in Supporting Information S1.
