# Supplementary material for: Oxygen Variability in the Offshore Northern Benguela Upwelling System From Glider Data
Source: J Geophys Res Oceans. 2022 Nov 5;127(11):e2022JC019063. doi: 10.1029/2022JC019063 (PMC9788292; doi:10.1029/2022JC019063)
Supplement: Supplementary file 1 — Supporting Information S1 [file JGRC-127-e2022JC019063-s001.pdf]

AGU PUBLICATIONS

Journal of Geophysical Research – Oceans

Supporting Information for

Oxygen variability in the offshore northern Benguela Upwelling System from glider data

Elisa Lovecchio[1], Stephanie Henson[1], Filipa Carvalho[1], Nathan Briggs[1]

[1]National Oceanography Centre, European Way, Southampton, U.K.

Contents of this file

Figures S1-S14, Tables S1,S2

Introduction

This file is a supplement to the manuscript "Oxygen variability in the offshore northern Benguela Upwelling System from glider data" and contains 14 figures and 2 tables.

Figures S1-S4 provide additional information about the analysis of the regional setting.
Figures S5-S13 provide additional information about the analysis of the glider data.
Figure S14 shows data calibration plots.
Table S1 provides information on the data used for the water mass identification (manuscript's Methods subsection 2.2).
Table S2 provides a full list of the datasets used in the manuscript.

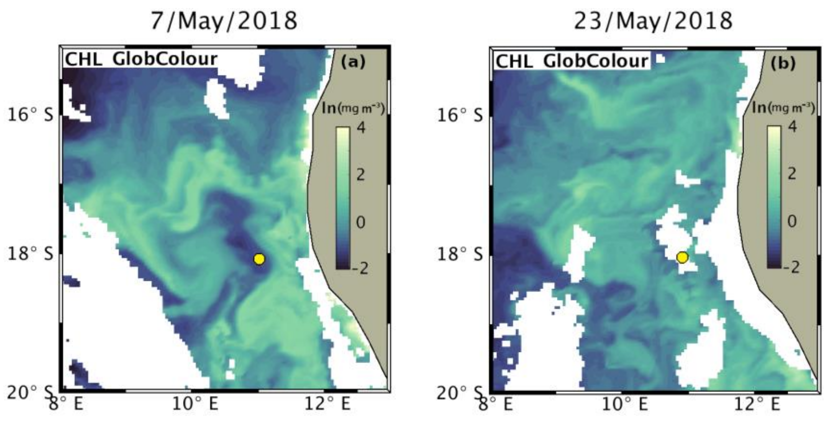

Figure S1. Chlorophyll [ln(mg m^{-3})] from satellite data for (a) 07 May 2018 and (b) 23 May 2018. White areas indicate missing data (cloud cover). The mean glider position is indicated by a yellow dot.

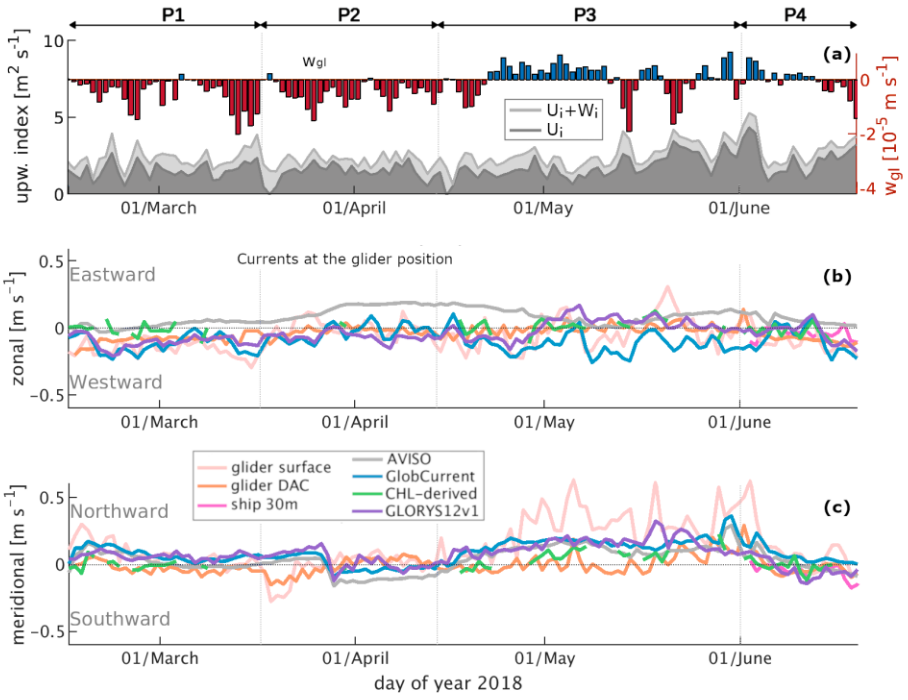

Figure S2. (a) Coastal upwelling index U_i and coastal Ekman pumping W_i (left y-axis, area plot) and Ekman pumping velocity at the glider position w_{gl} (right y-axis, bar plot) from ERA5 reanalysis. (b,c) Horizontal velocities at the glider position from satellite data (AVISO, GlobCurr), satellite chlorophyll derived data (CHL-der), model reanalysis data (GLORYS12V1), glider data both at the surface (glider SURF) and depth-averaged (DAC), and ship data for velocities at 30 m depth: (b) Zonal component, positive eastwards; (c) Meridional component, positive northwards. The duration of phases P1 to P4 is indicated by the horizontal arrows above subplot (a) and the dotted gray vertical lines.

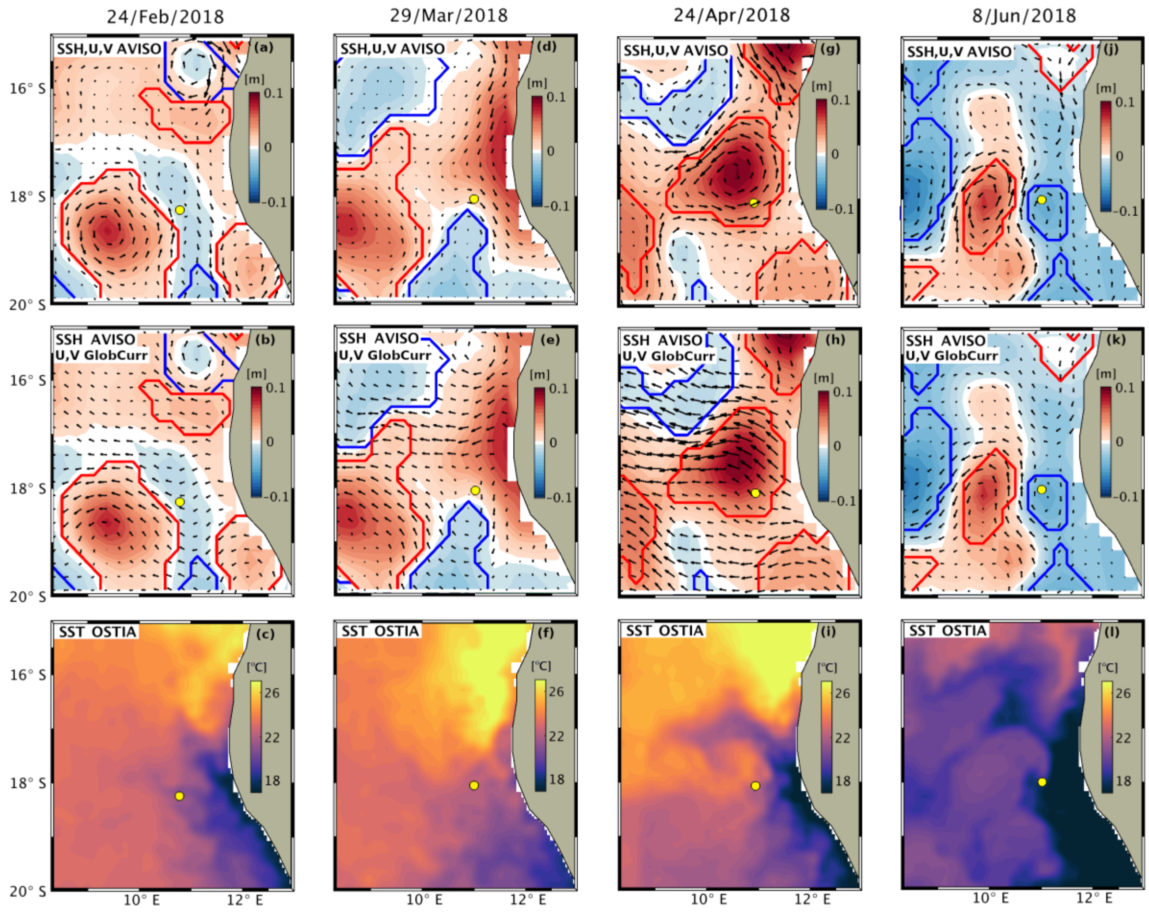

Figure S3. Regional setting from satellite data on 24/February (a-c), 29/March (d-f), 24/April (g-i), 08/June (j-l). First row: SSH with geostrophic velocities both from AVISO. Second row: SSH from AVISO with currents from GlobCurrent (CMEMS, 2022; Rio et al., 2014), the latter adding wind-driven modeled currents to the geostrophic currents derived from the AVISO SSH field. Third row: SST from L4 AVHRR-Ostia for comparison. Line contours on SSH in the first two rows indicate the identified cyclonic (blue) and anticyclonic (red) eddies. The mean daily glider position is indicated by a yellow dot.

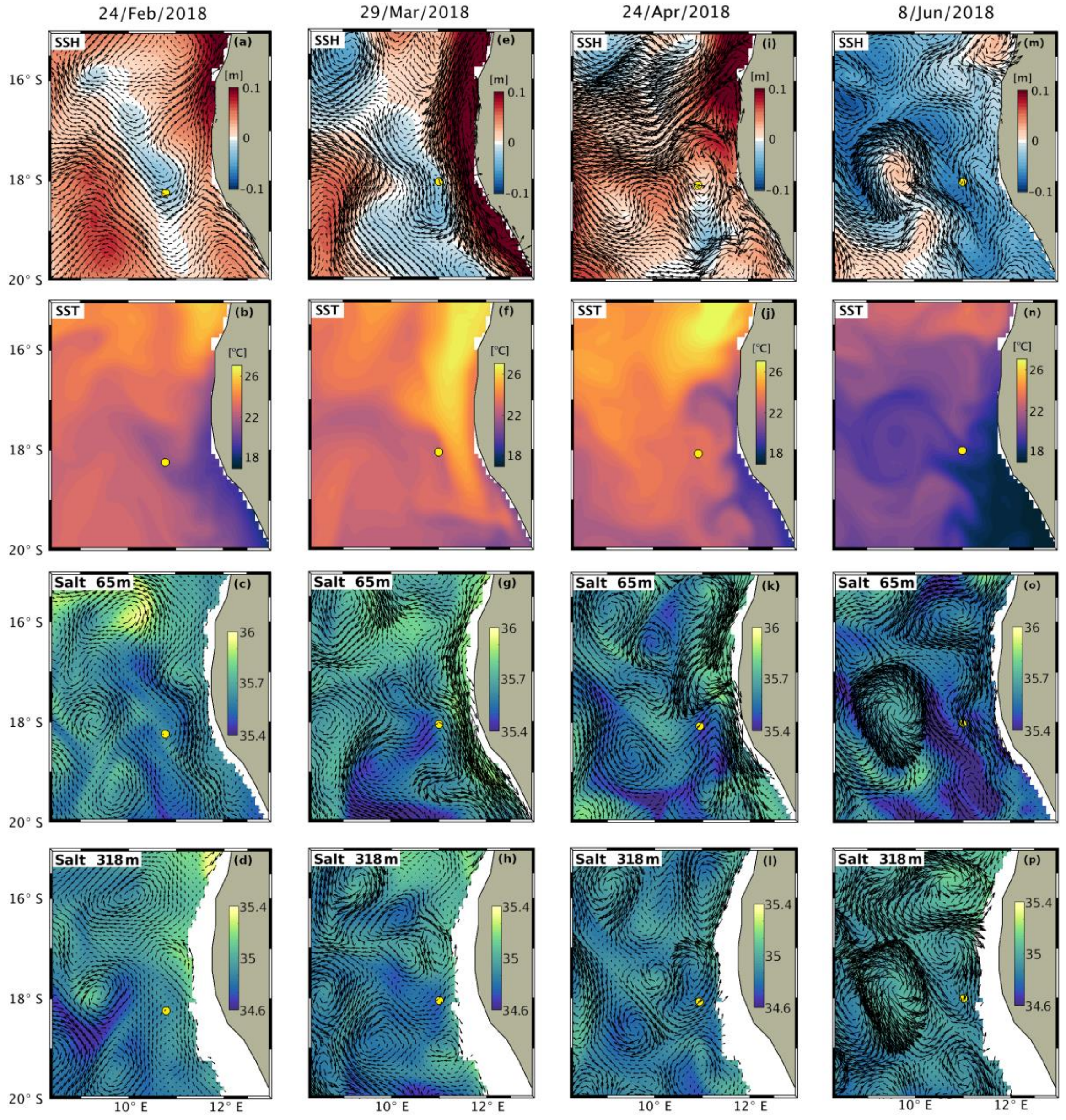

Figure S4. Regional setting according to model reanalysis data for 2018 from GLORYS12v1 (E.U. Copernicus Marine Service Information GLOBAL_REANALYSIS_PHY_001_030) on 24/February (a-d), 29/March (e-h), 24/April (i-l), 08/June (m-p). Note that the plotted days are the same used for Figure 2 in the manuscript. First row: sea surface height with surface currents. Second row: Sea surface temperature. Third row: currents and salinity at 65 m depth. Fourth row: currents and salinity at 318 m depth. The mean daily glider position is indicated by a yellow dot.

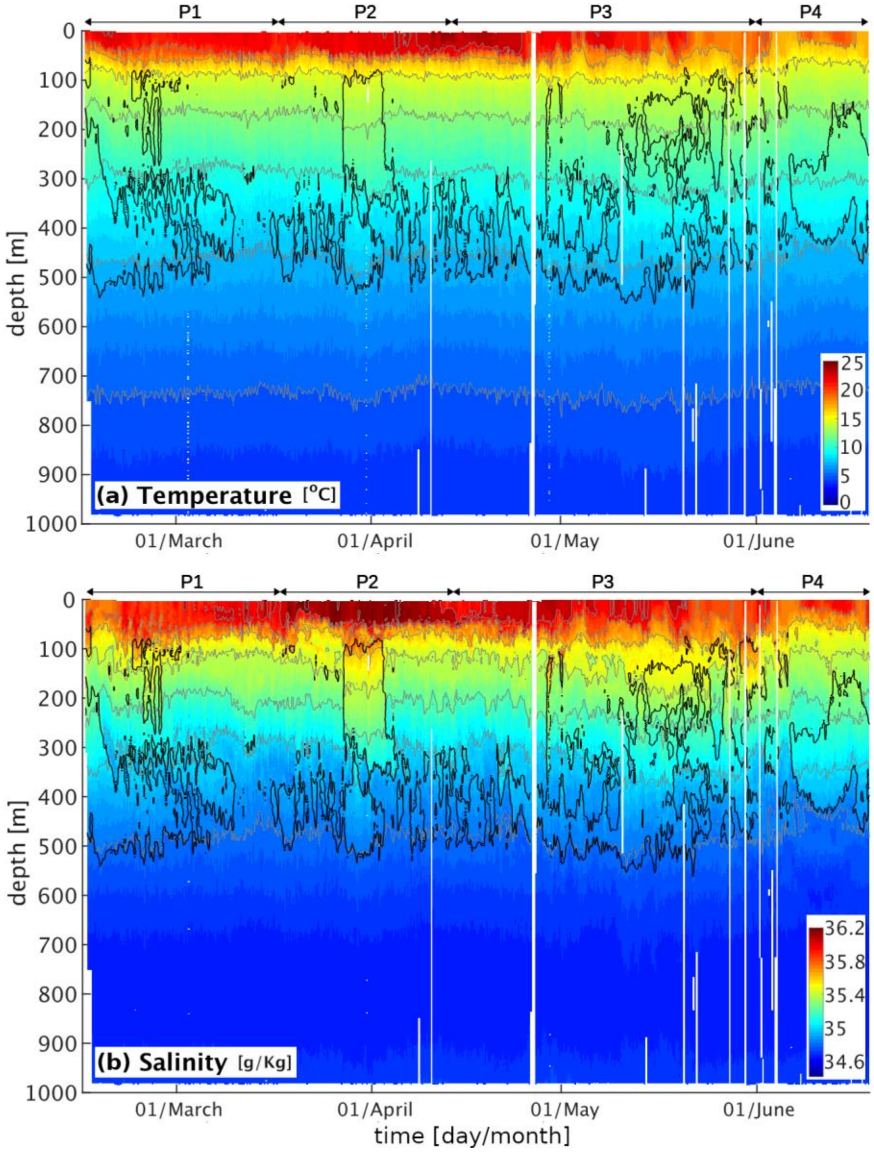

Figure S5. Full-depth glider transects: (a) conservative temperature, (b) absolute salinity. In gray, tracer isolines showing isolines of temperature every 2.5 ˚C from zero and isolines of salinity every 0.2 g kg^{-1} from 34.4 g kg^{-1}. In black, outer boundary of hypoxic regions (O_2 = 60 µmol kg^{-1}).

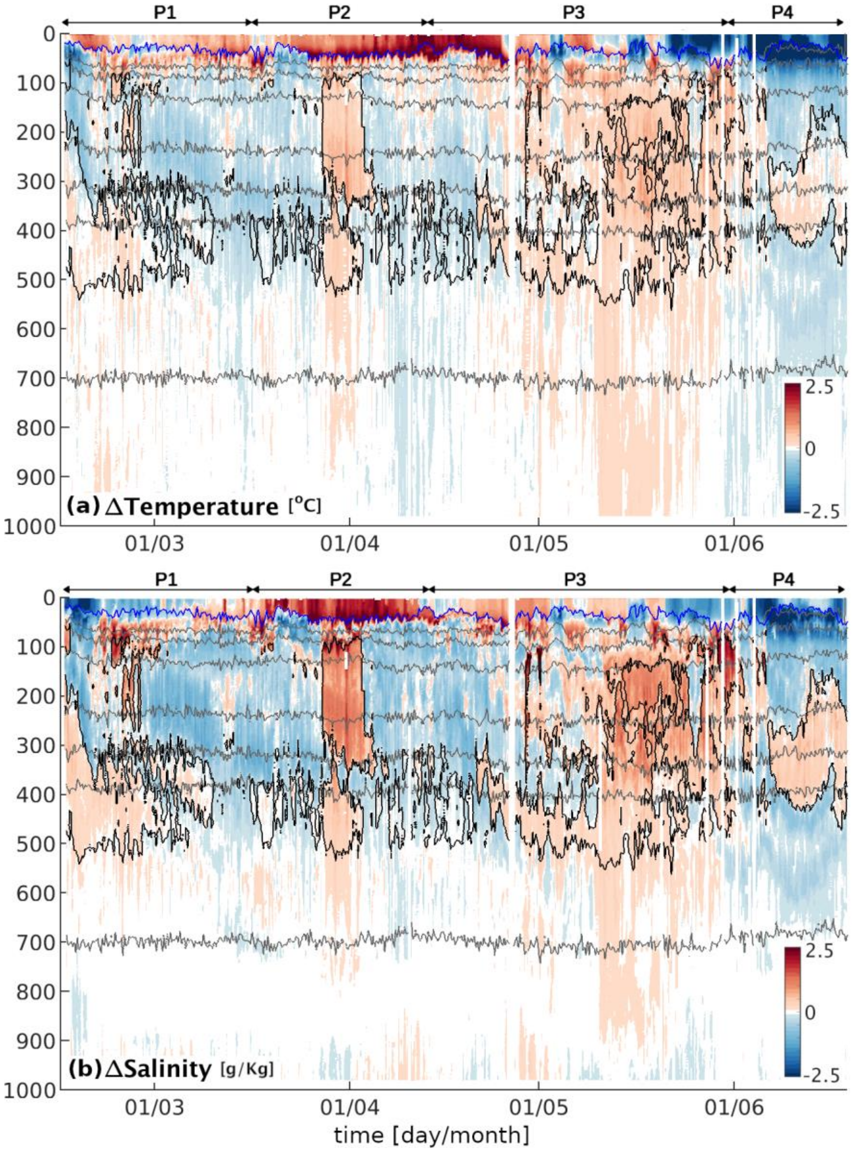

Figure S6. Full-depth glider transects: (a) Anomaly of conservative temperature, (b) Anomaly of absolute salinity. Gray lines: isopycnals for 26, 26.25, 26.5, 26.75, 26.9, 27 [kg m^{-3}]. Black lines: outer boundary of hypoxic regions (O_2 = 60 µmol kg^{-1}). Blue line: MLD [m].

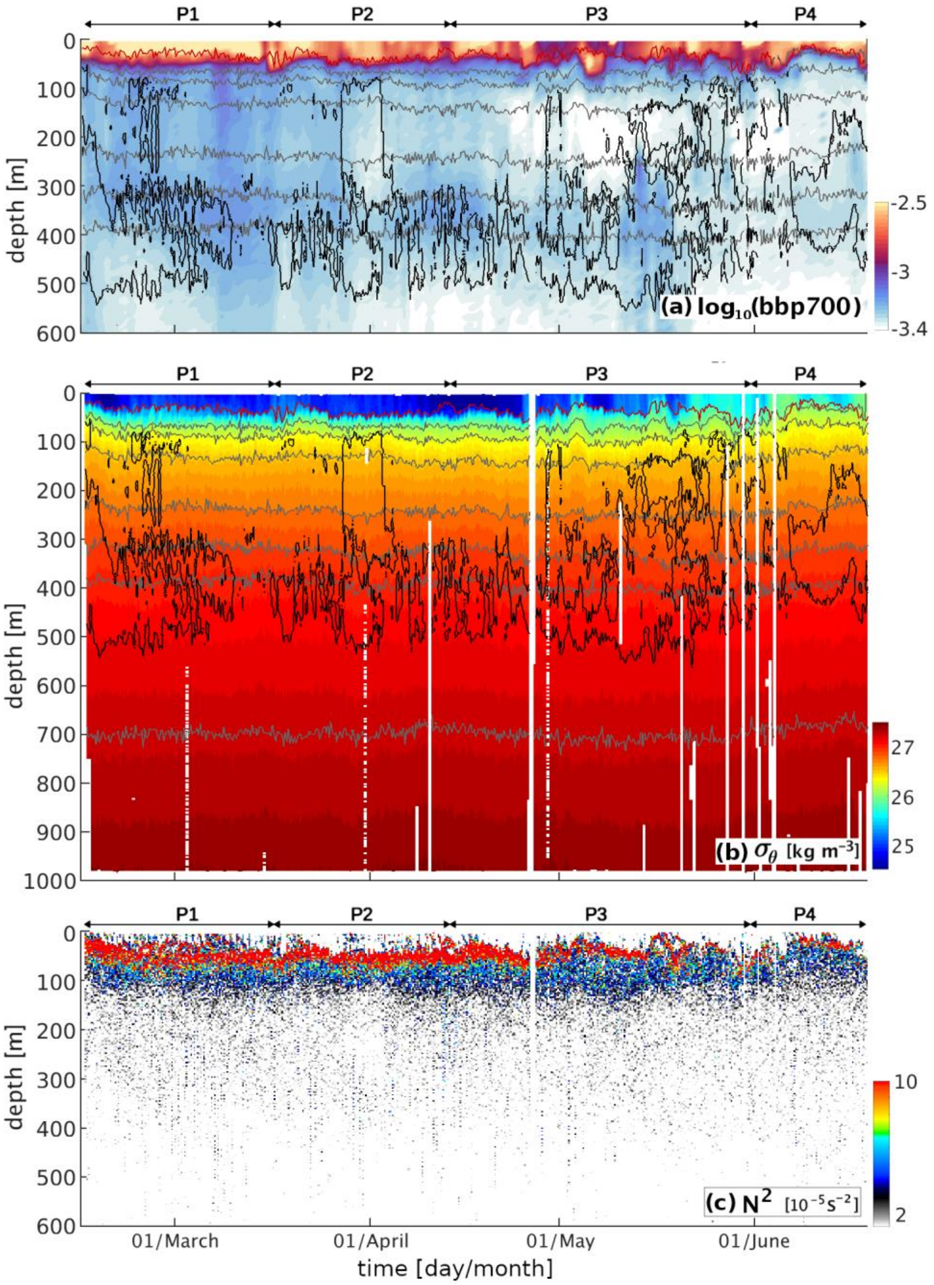

Figure S7. Glider transects: (a) Logarithm of backscattering at 700 nm, (b) Density. Gray lines: isopycnals for 26, 26.25, 26.5, 26.75, 26.9, 27 [kg m^{-3}]. Black lines: outer boundary of hypoxic regions (O_2 = 60 µmol kg^{-1}). Red line: MLD [m]. (c) N^2, no lines were added to better show the pattern.

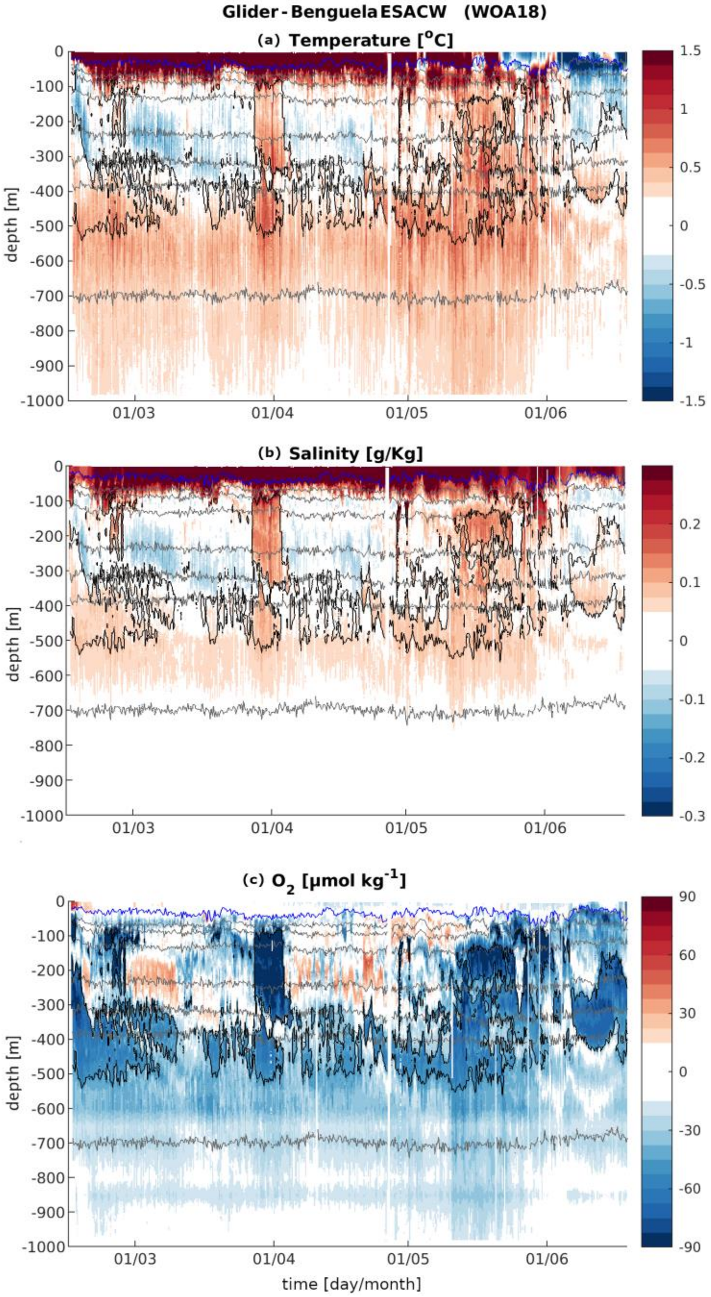

Figure S8. Full-depth difference between the glider data transects and the climatological mean profiles across the period February-June for the offshore Benguela, water properties from World Ocean Atlas 2018 (NOAA data set, 2018; Garcia et al., 2019) as described in the manuscript's Methods. (a) Conservative temperature T_c difference, (b) absolute salinity S_a difference and (c) oxygen O_2 concentration difference. White shading covers the range [-0.25, 0.25] ˚C for T_c, [-0.05, 0.05] kg m^{-3} for S_a, and [-15, 15] µmol kg^{-3} for O_2. Gray lines: isopycnals for 26, 26.25, 26.5, 26.75, 26.9, 27 [kg m^{-3}]. Black lines: outer boundary of hypoxic regions (O_2 = 60 µmol kg^{-1}). Blue line: MLD [m].

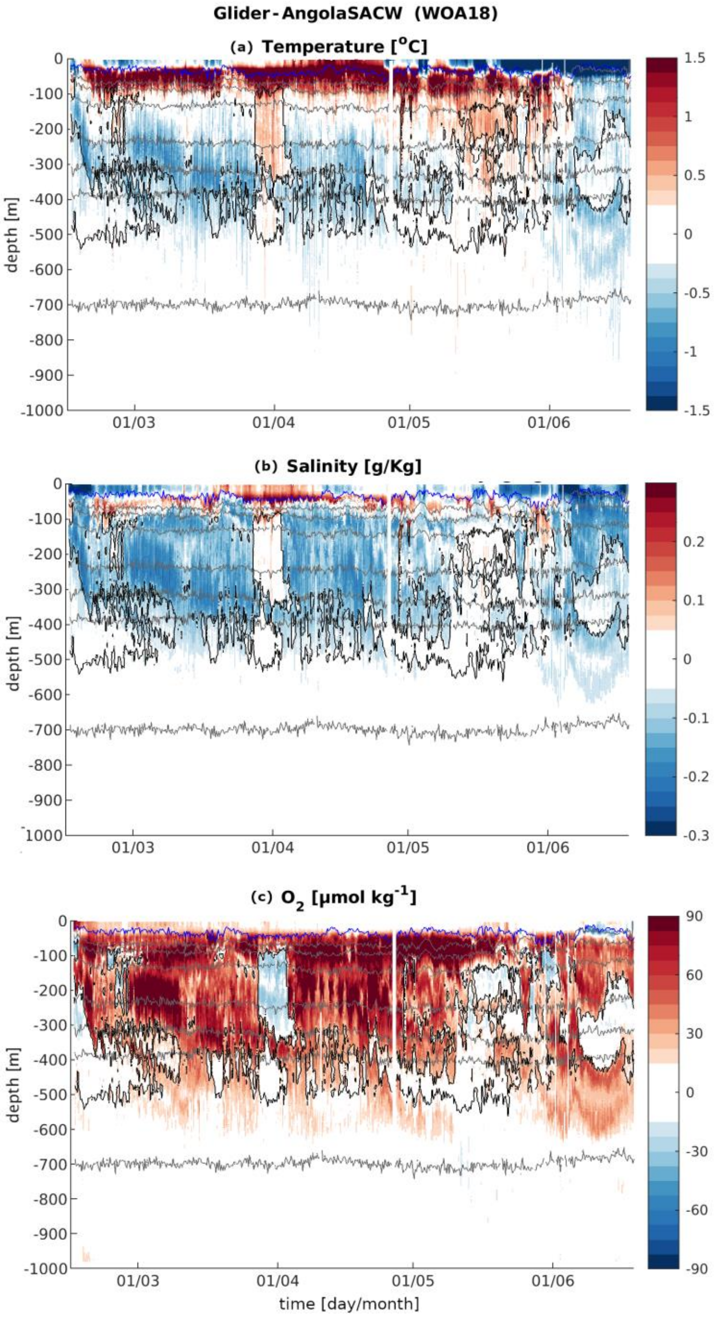

Figure S9. Full-depth difference between the glider data transects and the climatological mean profiles across the period February-June for the offshore Angola, water properties from World Ocean Atlas 2018 (NOAA data set, 2018; Garcia et al., 2019) as described in the manuscript's Methods. (a) Conservative temperature T_c difference, (b) absolute salinity S_a difference and (c) oxygen O_2 concentration difference. White shading covers the range [-0.25, 0.25] ˚C for T_c, [-0.05, 0.05] kg m^{-3} for S_a, and [-15, 15] µmol kg^{-3} for O_2. Gray lines: isopycnals for 26, 26.25, 26.5, 26.75, 26.9, 27 [kg m^{-3}]. Black lines: outer boundary of hypoxic regions (O_2 = 60 µmol kg^{-1}). Blue line: MLD [m].

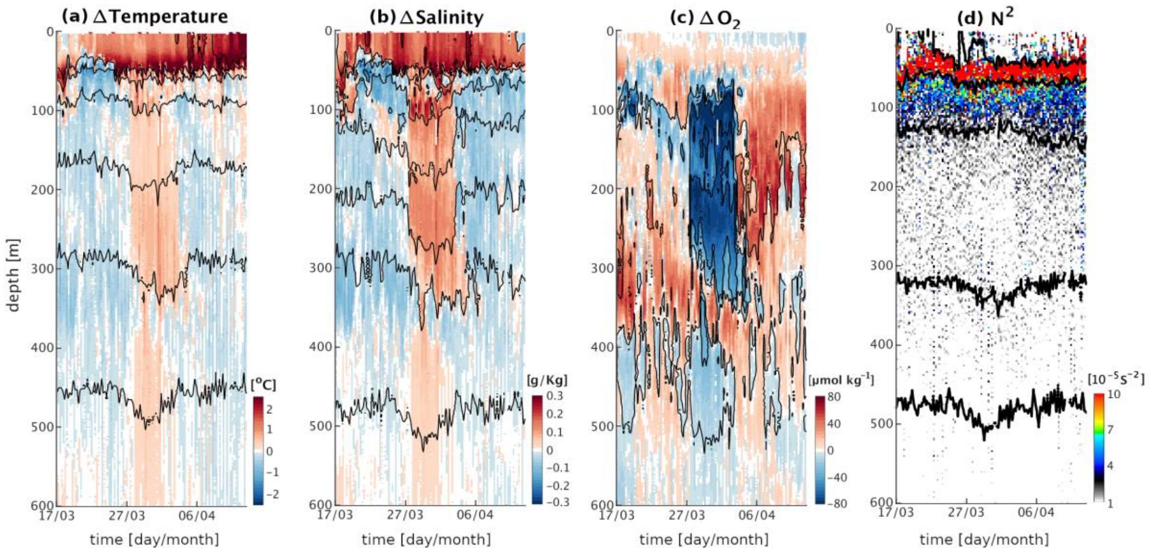

Figure S10. Glider transects and derived quantities for Phase 2 (17/March - 13/April): (a) conservative temperature anomaly and isolines, (b) absolute salinity anomaly and isolines, (c) oxygen anomaly and isolines, (d) Brunt-Väisälä frequency and isopycnal levels as thick black lines.

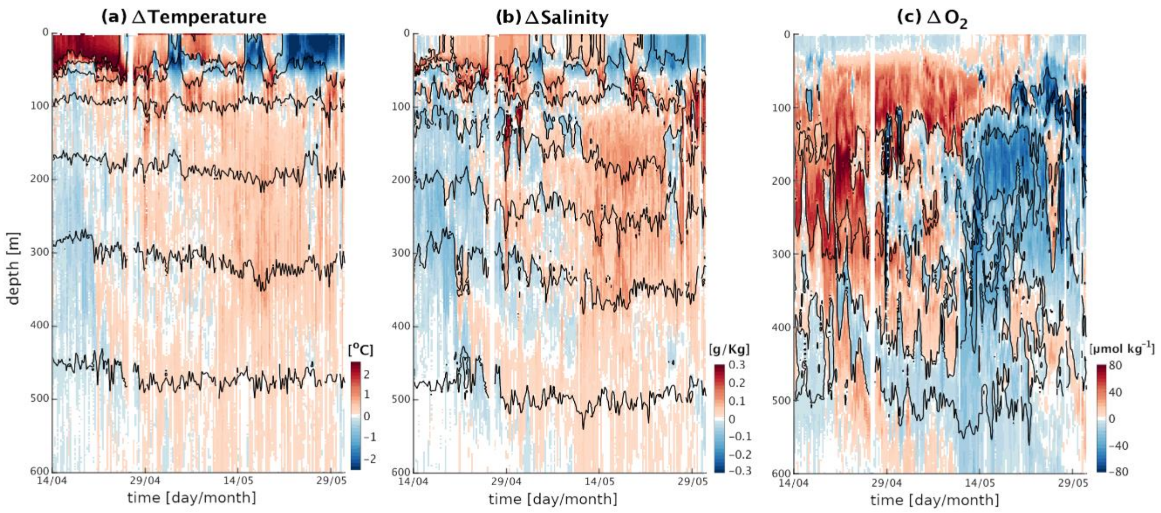

Figure S11. Glider transects and derived quantities for Phase 3 (16/April - 31/May): (a) conservative temperature anomaly and isolines, (b) absolute salinity anomaly and isolines, (c) oxygen anomaly and isolines.

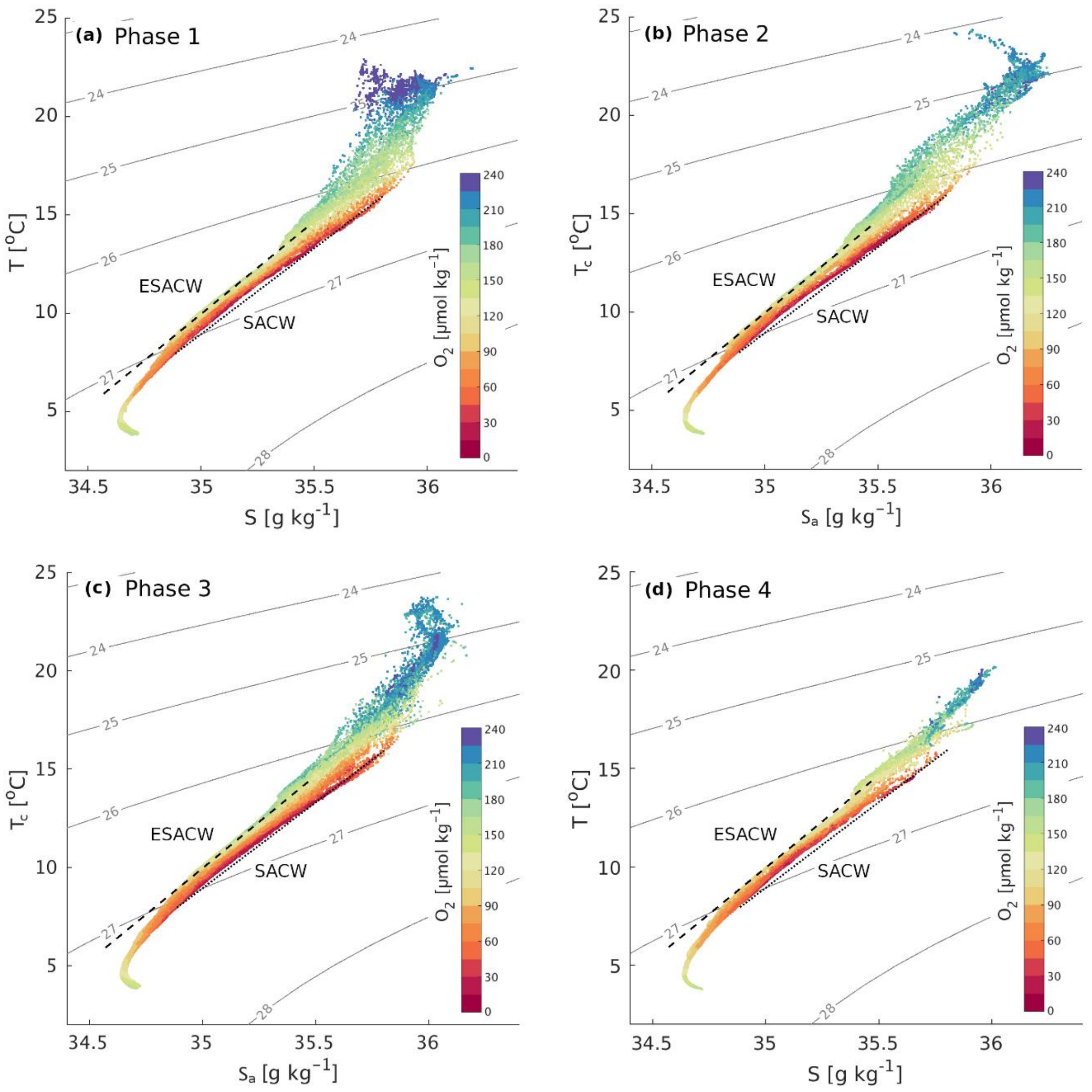

Figure S12. Conservative temperature (T_c) and absolute salinity (S_a) diagrams of the binned glider data during the four phases of the measurement period: (a) phase 1 (14/February - 16/March), (b) phase 2 (17/March - 13/April), (c) phase 3 (16/April - 31/May), and (d) phase 4 (01/June - 19/June). Dots are colored according to oxygen concentration. The characteristic T_c-S_a trends of SACW and ESACW are highlighted respectively by a dotted and a dashed black line.

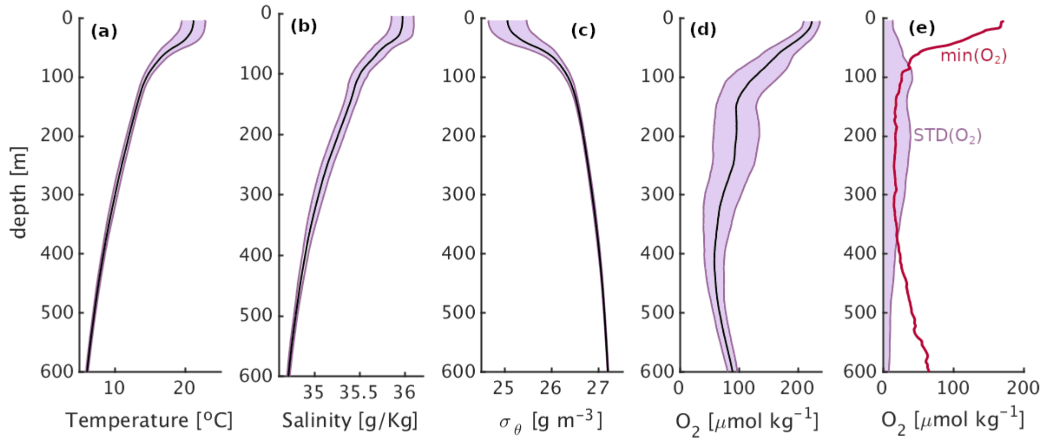

Figure S13. Mean vertical profiles (black lines) and color-shaded standard deviation (purple) for: (a) conservative temperature, (b) absolute salinity, (c) potential density, (d) oxygen. For oxygen only: (e) standard deviation (STD, purple) and minimum concentration (min, red) at each depth.

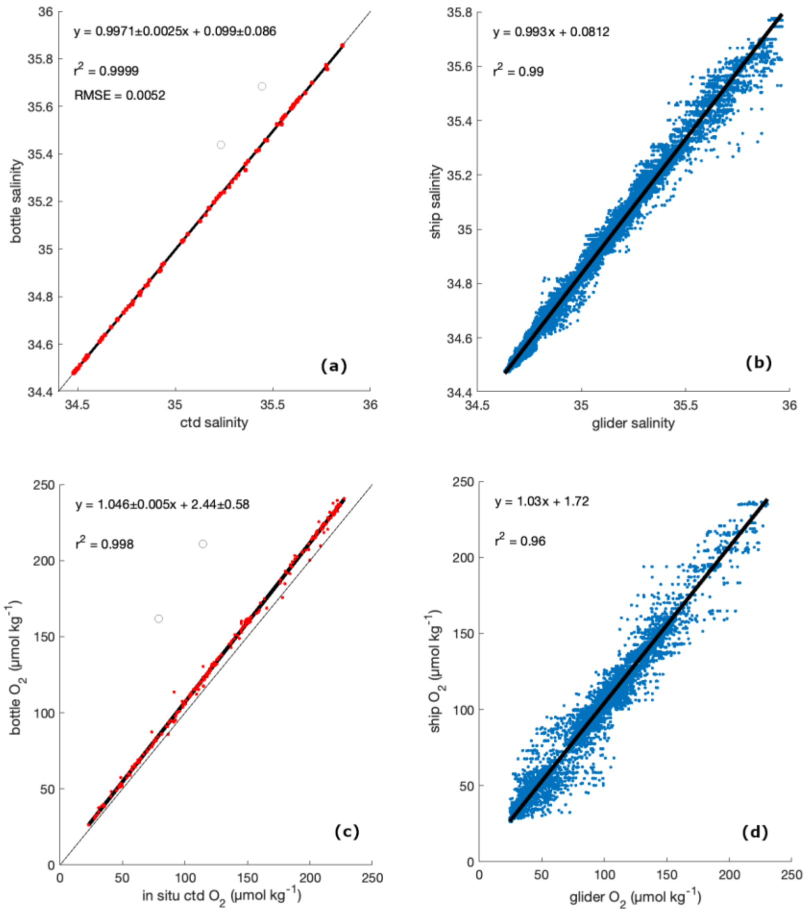

Figure S14: Bottle calibration of the ship sensors (left, red points) and calibration of the glider against the bottle-calibrated ship sensors (right, blue points) for salinity (a-b) and oxygen (c-d).

**SACW (Mohrholz et al., 2008)**						
T_i [˚C]	S_p [PSU]	lon [˚E]	lat [˚N]	p [dbar]	T_c [˚C]	S_a [g kg^{-1}]
8	34.7201	10	-22	400	7.95	34.89
16	35.6366	14	-18	50	15.97	35.80
**ESACW (Poole & Tomczak, 1999)**						
T_i [˚C]	S_p [PSU]	lon [˚E]	lat [˚N]	p [dbar]	T_c [˚C]	S_a [g kg^{-1}]
5.9559	34.4069	16	-36	400	5.92	34.57
14.4000	35.3016	18	-30	50	14.37	35.47

Table S1. Supplement table to Methods' subsection 2.2 "Water mass identification". Water mass definition for SACW and ESACW according to literature (in-situ temperature T_i, practical salinity S_p) and after conversion to the units of conservative temperature T_c and absolute salinity S_a used in the current manuscript. We used average longitude (lon) and latitude (lat) positions for the water masses and assumed a pressure (p) of 400 dbar for the deeper values and of 50 dbar for the shallower values. Please, note that our calculations showed that the precise choice of longitude and latitude has a negligible impact on the results.

Variables	Source	Resolution	Reference / DOI
Temperature, Salinity, Oxygen *(gridded in-situ)*	World Ocean Atlas 2018	1˚ x 1˚, monthly mean climatology (1955-2017)	NOAA data set, 2018; Garcia et al., 2019
Sea surface height, surface geostrophic currents *(satellite)*	AVISO DUACS	0.25˚ x 0.25˚, daily means	CMEMS data, 2022 doi:10.48670/moi-00148
Surface currents *(satellite+model)*	COPERNICUS-GlobCurrent REP L4	0.25˚ x 0.25˚, daily means	Rio et al., 2014 doi:10.48670/moi-00050
Sea surface temperature *(satellite)*	Ostia reprocessed	0.05˚x 0.05˚, daily means	Good et al., 2020 doi:10.48670/moi-00168
Temperature, salinity, currents *(model reanalysis)*	GLORYS12v1 reanalysis	1/12° x 1/12°, daily means	Fernandez & Lellouche, 2018; GLOBAL_REANALYSIS_PHY_001_030
Surface chlorophyll *(satellite)*	GlobColour L3	4 km, daily means	CMEMS, 2020 doi:10.48670/moi-00100
Surface chlorophyll *(satellite)*	MODIS Aqua, MODIS Terra, VIIRS	1 km, daily	NASA-OBPG 2018a,b,c
Wind stress *(model reanalysis)*	ERA5 reanalysis	0.25˚x0.25˚, daily means	Hersbach et al. 2020

Table S2. List of the online datasets used in the manuscript, of their properties and references. Full references for these citations can be found in the main manuscript's bibliography.
